# A grape seed extract maternal dietary supplementation in reproductive hens reduces oxidative stress associated to modulation of plasma and tissue adipokines expression and improves viability of offsprings

**DOI:** 10.1371/journal.pone.0231131

**Published:** 2020-04-13

**Authors:** Alix Barbe, Namya Mellouk, Christelle Ramé, Jérémy Grandhaye, Christophe Staub, Eric Venturi, Marine Cirot, Angélique Petit, Karine Anger, Marine Chahnamian, Patrice Ganier, Olivier Callut, Estelle Cailleau-Audouin, Sonia Metayer-Coustard, Antonella Riva, Pascal Froment, Joëlle Dupont

**Affiliations:** 1 INRAE UMR85 Physiologie de la Reproduction et des Comportements, Nouzilly, France; 2 CNRS UMR7247 Physiologie de la Reproduction et des Comportements, Nouzilly, France; 3 Université François Rabelais de Tours, Tours, France; 4 IFCE, Nouzilly, France; 5 INRAE - Unité Expérimentale de Physiologie Animale de l’Orfrasière UEPAO 1297, Nouzilly, France; 6 INRAE - Unité Expérimentale du Pôle d’Expérimentation Avicole de Tours UEPEAT, 1295, Nouzilly, France; 7 INRAE, UMR0083 Biologie des Oiseaux et Aviculture, Nouzilly, France; 8 INDENA, Tours, France; University of Illinois, UNITED STATES

## Abstract

In reproductive hens, a feed restriction is an usual practice to improve metabolic and reproductive disorders. However, it acts a stressor on the animal. In mammals, grape seed extracts (GSE) reduces oxidative stress. However, their effect on endocrine and tissue response need to be deepened in reproductive hens. Here, we evaluated the effects of time and level of GSE dietary supplementation on growth performance, viability, oxidative stress and metabolic parameters in plasma and metabolic tissues in reproductive hens and their offsprings. We designed an *in vivo* trial using 4 groups of feed restricted hens: A (control), B and C (supplemented with 0.5% and 1% of the total diet composition in GSE since week 4, respectively) and D (supplemented with 1% of GSE since the hatch). In hens from hatch to week 40, GSE supplementation did not affect food intake and fattening whatever the time and dose of supplementation. Body weight was significantly reduced in D group as compared to control. In all hen groups, GSE supplementation decreased plasma oxidative stress index associated to a decrease in the mRNA expression of the NOX4 and 5 oxidant genes in liver and muscle and an increase in SOD mRNA expression. This was also associated to decreased plasma chemerin and increased plasma adiponectin and visfatin levels. Interestingly, maternal GSE supplementation increased the live body weight and viability of chicks at hatching and 10 days of age. This was associated to a decrease in plasma and liver oxidative stress parameters. Taken together, GSE maternal dietary supplementation reduces plasma and tissue oxidative stress associated to modulation of adipokines without affecting fattening in reproductive hens. A 1% GSE maternal dietary supplementation increased offspring viability and reduced oxidative stress suggesting a beneficial transgenerational effect and a potential use to improve the quality of the progeny in reproductive hens.

## Introduction

Since several decades, the growth rate of reproductive hens has strongly increased due to genetic progress and management strategies [1]. The increasingly heavy and fatty animals developed metabolic and musculoskeletal disorders [2]. In order to avoid an excessive fattening and its associated disorders, a strict feed restriction is applied at a very early age in broiler breeder hens. Thus, improvements in metabolic and reproductive performances were shown in reproductive hens with restricted diet [3]. Nowadays, restricted diet is currently practiced in broiler breeding but this practice induces stress [4] and aggressive behaviours [5]. Moreover, the genetic selection towards large breast muscles and fast growth rates increased the susceptibility to oxidative stress for domestic birds like broilers. Poultry meat has also been traditionally recognized as highly sensitive to oxidative processes due to the high unsaturation degree of the muscle lipids [6].

The oxidative stress results from an imbalance between the production of free radicals including reactive oxygen species (ROS) and the ability of the organism to counteract their harmful effects through neutralization by antioxidants [7]. In farm animals, several reports suggested that oxidative stress may be involved in many disorders affecting animal production and welfare [8]. In chicken, oxidative stress induced by alterations of environmental conditions such as heat reduces growth and meat quality [9]. Supplementation of natural antioxidant compounds to chicken food has been proposed as a means for reducing the oxidative stress-induced adverse effects [10–13]. Polyphenols are bioactive phytochemical compounds and mostly studied due to their antioxidant properties. In mammals, many studies have suggested that polyphenols’ antioxidant activity may improve the well-being of living organisms and protect against several diseases [14]. One of the polyphenols’ sources is the grape seed from waste products of the winery and grape juice industry [15]. More specifically, grape seed extracts (GSE) from the seeds of *Vitis vinifera*, are sources of flavonoids, particularly proanthocyanidins (POC) that are known to have beneficial effects on oxidative stress and on many metabolic disorders including insulin resistance [16], increased adiposity [17], and inflammation [18] in mammals. Moreover, it has been reported that some of these GSE effects could be associated to modulations of plasma adipokines such as adiponectin [19]. In mammals, different studies have demonstrated that maternal consumption of polyphenols including GSE during prenatal periods can positively affect the health of the offspring [20–26].

In broiler chickens, GSE dietary supplementation has been demonstrated to be an immunostimulant agent [27, 28] and to improve fatty acid profile in liver [29]. In hens, it was also able to modulate egg quality and composition and laying performance [30, 31], (supplementation for 5 weeks) and to prevent the ovarian aging process [32]. However, the effects of chronic GSE dietary supplementation on oxidative stress, growth performance and metabolic parameters including plasma adipokines have never been investigated in hens. In addition, to the best of our knowledge, transgenerational effects of grape seed extracts have not been examined yet in hens. So, the present study was designed to evaluate the effects of time and level of maternal GSE dietary supplementation on growth performance, viability, oxidative stress and metabolic parameters in plasma and metabolic tissues in both reproductive hens and their offsprings.

## Materials and methods

### Ethical issues

All experimental procedures were performed in accordance with the French National Guidelines for the care and use of animals for research purposes (certificate of authorisation to experiment on living animals APAFIS number 10237-201706151202940v3), Ministry of Agriculture and Fish Products, and favourable notice of ethics committee of Val de Loire N°19).

### Animals

Three hundred and twenty-four broiler breeder females chicks from Hendrix Genetics (Saint Laurent de la Plaine, France) were studied from day 1 to 40 weeks of age. Animals were divided in groups of 10 to 11 birds in 32 pens, each pen with an area of 3 m^2^. The animals were reared at “Pôle Expérimental Avicole de Tours” (INRA, Nouzilly, France) according to the conventional conditions of breeding: 24h of light per day on arrival, then the day length was reduced to approximately 8h at the 1^st^ week, kept constant until the age of photostimulation (21^st^ week) and then a gradual increase until reaching 14h of light per day at the end of study (40^st^ week). At 40 weeks, all animals were killed by electrical tunning and bled out as recommended by the ethical committee.

### Diets composition

From one to 28 days (week 4) of age, female breeder chicks were distributed into two treatment groups: the first group (n = 252, control) received *ad libitum* starting diet and the second group (D group, n = 72) received the same diet supplemented with GSE at 1% of total diet (S1 Fig). From 28 (week 4) to 280 days (week 40) of age, all animals received a restricted diet according to Hendrix Genetic recommendation, the D group still received 1% GSE supplementation and the control group was subdivided into three groups: one with 0.5% GSE supplementation (group B, n = 80), one with 1% GSE supplementation (group C, n = 80) and one without GSE (group A, n = 92). During this period, animals received three different diets: growing (from week 4 to 17), before laying (from week 18 to 23) and laying (from week 24 to 40) (S1 Fig S1 Table). The GSE supplement was provided by INDENA (Tours, France). The determination of procyanidins of GSE was analysed by HPLC (High Performance Liquid Chromatography) by INDENA and showed that the most important component was the procyanidins (> 90%).

### Determination of food intake

From one to 28 days, remaining feed was weighed each day in control and D group. From 28 (week 4) to 280 days (week 40) of age, the restricted animals in each group ate their whole ration.

### Determination of live body weight, fattening level and feed conversion

The fasted hens were weighed every three weeks using an automated balance from Grosseron (B146782-ENTRS 8201i-1S, Coueron, France) with a precision of 0.1g. The fattening level of the animals were assessed every 3 weeks by ultrasonographic examination (MyLab 30 Gold Vet, Hospimedi France, Saint Crépin-Ibouvillers) as previously described [33]. Feed conversion was determined as the ratio between the total food intake during a fixed period (starting, growing and before laying) and the total gain in body weight during this same fixed period.

### Plasma biochemical parameters

Blood samples were collected from the occipital sinus into heparin tubes at weeks 4, 6, 12, 21, 24, 30, 33 and 40 (10 animals/group). Plasma was recovered after centrifugation at 5000 g for 10 min at 4°C and stored at -20°C until use. Plasma concentration of glucose, triglycerides, uric acid, calcium and phospholipids were determined by enzymatic assay using specific kits: glucose GOD-POD (MG981780, ThermoScientific, Asnières sur Seine, France), triglycerides (MG981786, ThermoScientific, Asnières sur Seine, France), uric acid AOX (MG981788, ThermoScientific, Asnières sur Seine, France), calcium (Biolabo SAS, Maizy, France) and phospholipids assays (Biolabo SAS, Maizy, France), respectively. The measurements were performed according to the manufacturer’s protocol.

### Tissues collection

At 40 weeks, all animals were killed by cervical dislocation and abdominal adipose tissue, liver and pectoral muscles from 10 animals per group were collected, immediately frozen in liquid nitrogen and stored at -80°C. To confirm the fattening level determined by ultrasonographic examination abdominal adipose tissues were dissected and weighted for all animals of each group.

### TAS, TOS and OSI determination

TAS (total antioxidant status) usually used to measure the overall antioxidant status of the body [34] was determined using a commercial kit supplied by Randox (Crumlin, UK) according to the manufacturer’s instructions. In this method, metmyoglobin is converted into ferrylmyoglobin in the presence of iron ions. The result of the reaction between ferrylmyoglobin with the Randox ABTS reagent was a green product, the absorbance of which was measured at 600 nm. The TOS (total oxidant status) concentration usually used to estimate the overall oxidation state of the body [35] was determined using the commercial kit Per-OX TOS/TOC (Immune Diagnostics, Bensheim, Germany). The reaction of peroxidase with lipid hydroperoxides led to the production of reduced phospholipid products of green color, which changes to yellow upon addition of the stop reagent. Absorbance was measured at 450 nm. The index of oxidative stress (OSI) was calculated based on the formula = TOS/TAS x 100 [36]. It is a comprehensive measurement of TAS and TOS.

### Plasma adipokines assays

The concentration of three adipokines, visfatin (NAMPT), adiponectin (ADIPOQ) and chemerin (RARRES2) were determined in the plasma using chicken specific ELISA kits as previously described [33]. MBS269004 (sensitivity 5 pg/mL), MBS016609 (sensitivity 0.1 μg/mL) and MBS738819 (sensitivity 0.1 ng/mL), were used for NAMPT, ADIPOQ and RARRES2, respectively (My BioSource, San Diego, USA). The experiment was performed following the manufacturer’s protocol with an intra-assay coefficient of variation ≤ 8%, < 10% and < 5.6, respectively. The absorbance was measured at 450 nm and then compared with reference values.

### mRNA expression of lipid metabolism factors in abdominal adipose tissue, adipokines and their receptors in abdominal adipose tissue, liver and pectoralis major muscle

For abdominal adipose tissue, pectoralis major muscle and liver, total RNA was extracted by homogenization in the TRIzol tissue reagent using an Ultraturax, according to the manufacturer’s recommendations (Invitrogen, by Life Technologies, Villebon sur Yvette, France). The cDNA was generated by reverse transcription of total RNA (2 μg) in a mixture comprising 0.5 mM of each deoxyribonucleotide triphosphate (dATP, dTTP, dGTP, dCTP), 2 M of RT Buffer, 15 μg/μL of oligodT, 0.125 U of ribonuclease inhibitor, and 0.05 U of Moloney murine leukemia virus reverse transcriptase (MMLV) for one hour at 37°C. Real-time PCR was performed using the MyiQ Cycle Device (Bio-Rad, Marnes-la-Coquette, France), in a mixture with SYBR Green Supermix 1X Reagent (Bio-Rad, Marnes-la-Coquette, France), 250 nM specific primers (Invitrogen by Life Technologies, Villebon-sur-Yvette, France) and 3 μL of cDNA diluted five-fold) for a total volume of 11 μL. The samples were duplicated on the same plate as previously described [33]. The primers used are shown in S2 Table. For each gene, the relative abundance of transcription was determined by the calculation of e^-ct^. Then, the relative expression of the gene of interest was related to the relative expression of the geometric mean of the two reference genes (*RPL15* and *β actin*).

### Determination of the body weight and mortality in the offsprings

The semen of 48 cocks (Cobb500) was collected and pooled to form a single sample. An artificially insemination was performed with 2x10^8^ spermatozoa at the 28^th^ week and 33^rd^ week. Eggs were collected and incubated during 21 days. The offsprings (n = 1173, 1159, 1114 and 804 chicks from hens of groups A, B, C and D, respectively) were weighed at hatching and at day 10 (D10). They were all fed with the same starting diet unsupplemented with GSE. Each day the number of dead animals was noted and the mortality level was calculated from hatching to day 10. At hatching and day 10, at least 10 animals from hens of each group (A to D) were killed and then tissues (abdominal adipose tissue, pectoralis major muscle and liver) were collected.

### Statistical analysis

The results are represented as mean ± SEM, with a level of significance less than 0.05 (*P < 0.05). Different letters indicate significant differences (P < 0.05). SAS Software (version 9.3, Cary, USA) was used for all analyses. An analysis of variance using repeated measurements (Proc.Mixed procedure) was used to compare the mean values for live body weight, fat thickness, feed conversion, plasma parameters (TAS, TOS, oxidative stress index, triglycerides, phospholipids, glucose, calcium, uric acid, and adipokines (RARRES2, ADIPOQ and NAMPT) for the different groups of hens (A to D) and for live body weight of chicks (at hatching and 10 days of age). Two factors were analysed: the time of GSE supplementation (from hatch (one day of age, D group) compared from 28 days (week 4, B and C groups)) and a diet effect. If the time of GSE supplementation was significant, we analysed separately the diets A and D, and A,B and C and if not we compared the whole A,B,C and D groups. For each comparison, each period (starting, growing, before laying and laying) was analysed separately. An analysis of variance (Proc.GLM procedure) was used to compare the average levels of mRNA expression data among the different groups. A chi-square test was used for analysis of percentage mortality of chicks from different groups of hens. A Pearson test was used to analyse correlations between plasma adipokine levels, live body weight and oxidative stress parameters. The correlation was noted ‘r’ and the P value was considered significant if *P* < 0.05.

## Results

### Effects of GSE supplementation on food intake, body weight, fattening level and feed conversion

**Fig 1:**
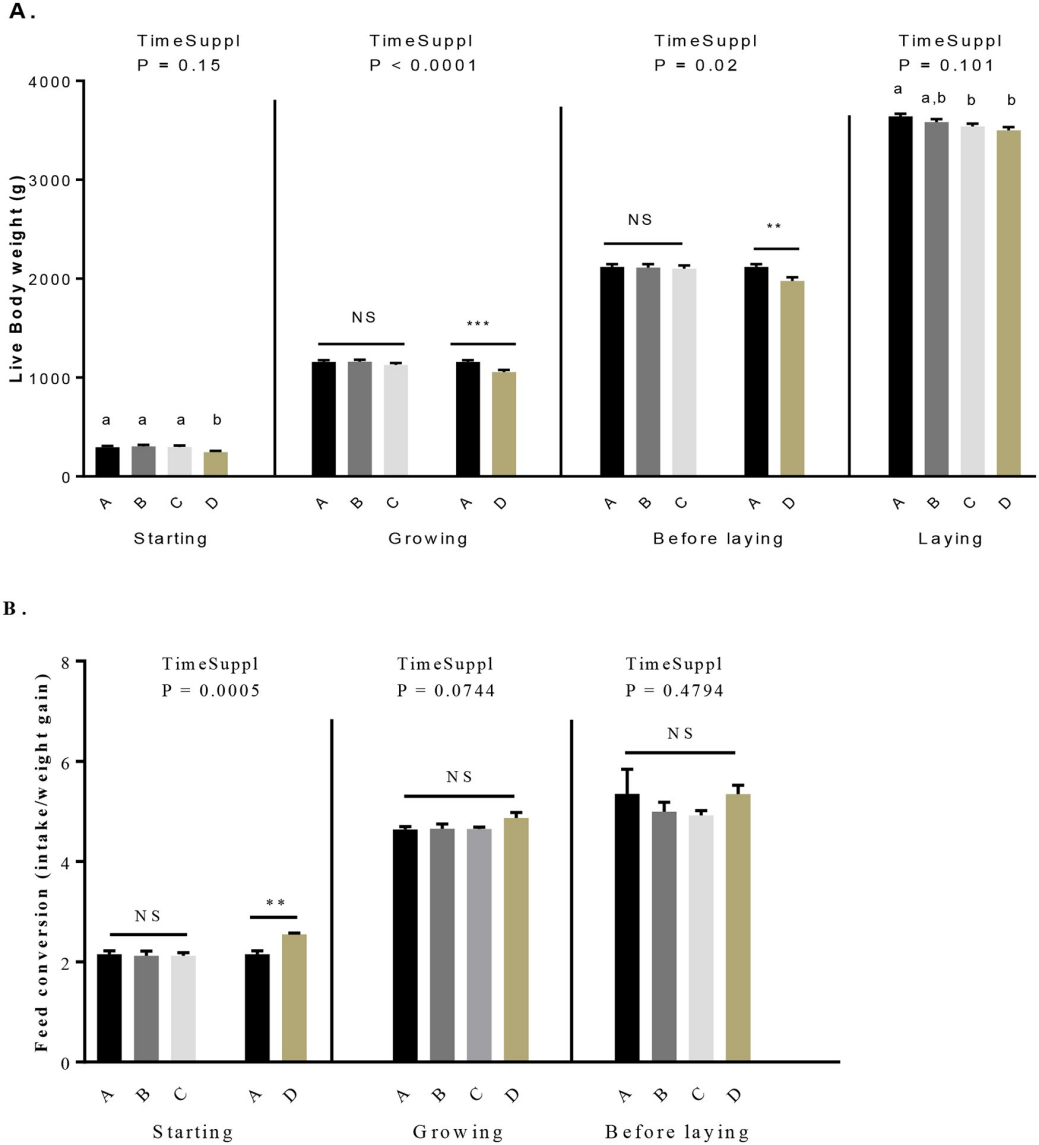
Live body weight (A) and feed conversion (B) in broiler hens fed with different concentrations of GSE dietary supplementation. A: animals fed with control diet without GSE supplementation (n = 92), B and C: animals supplemented with GSE at 0.5% and 1% of the total diet composition, respectively, starting at 4 week-old until 40 week-old (n = 80), and D: supplementation at 1% of the total diet composition starting at hatch until 40 week-old (n = 72). The starting period corresponds to the hatching until the 4th week, the growing period corresponds to the 4th week until 17th week, the « before laying » period corresponds to the 18th week to the 23rd week and the laying period corresponds to the 24th week until the 40th week. Results are presented as lsmeans ± SEM. *P* values of the effects of the stage when the supplementation is applied (TimeSuppl) and diet were considered as significant if *P* < 0.05. Different individual letters (a, b and c) indicate a significant effect of the diet. ** *P* < 0.005 and *** *P* < 0.001 (diet effect).

**Fig 2:**
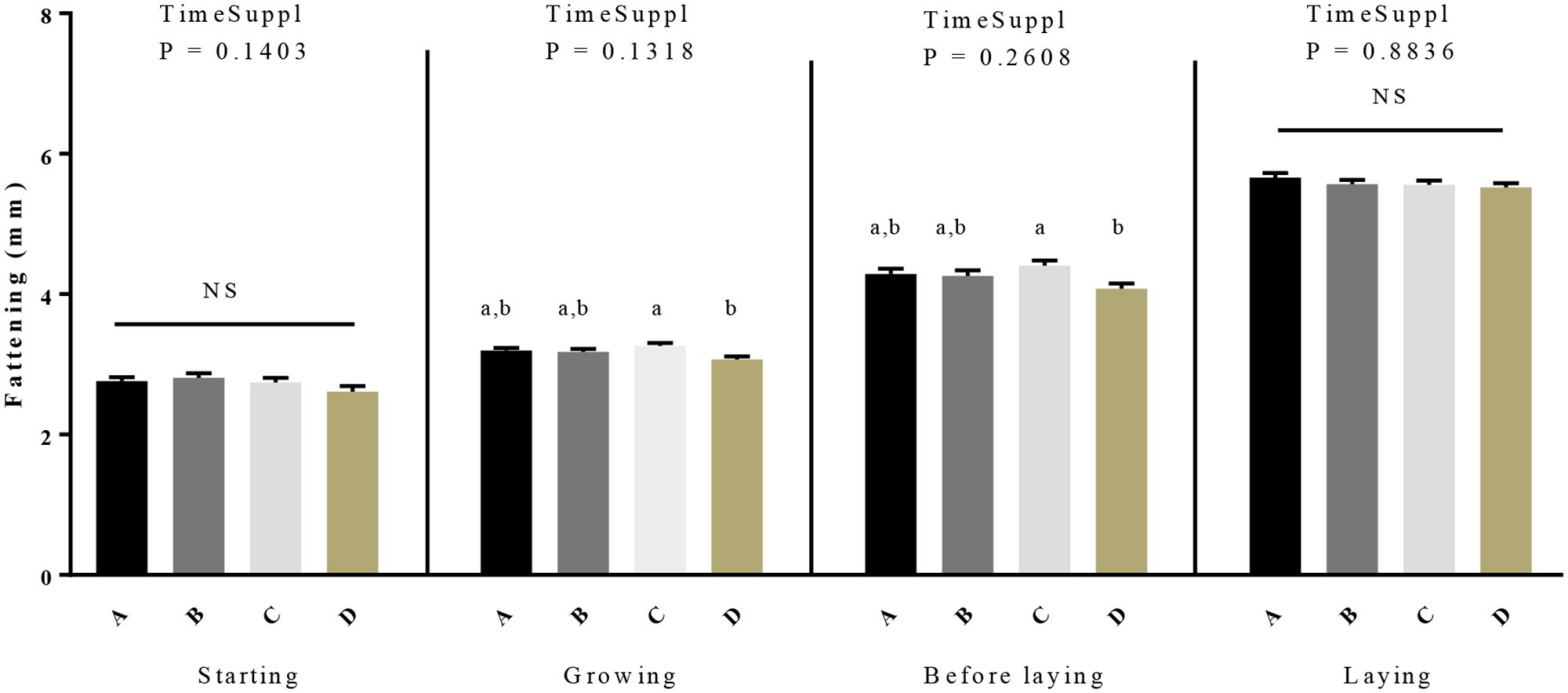
Fattening level in broiler hens fed with different concentrations of GSE dietary supplementation. The fattening was assessed by ultrasound examinations. A: animals fed with control diet without GSE supplementation (n = 92), B and C: animals supplemented with GSE at 0.5% and 1% of the total diet composition, respectively, starting at 4 week-old until 40 week-old (n = 80), and D: supplementation at 1% of the total diet composition starting at hatch until 40 week-old (n = 72). The starting period corresponds to the hatching until the 4th week, the growing period corresponds to the 4th week until 17th week, the « before laying » period corresponds to the 18th week to the 23rd week and the laying period corresponds to the 24th week until the 40th week. Results are presented as lsmeans ± SEM. *P* values of the effects of the stage when the supplementation is applied (TimeSuppl) and diet were considered as significant if *P* < 0.05. Different individual letters (a, b and c) indicate a significant effect of the diet.

From 1 to 28 days, the daily food intake was similar between control and D group and from 29 to 280 days there was no food left in the feeder for all restricted animals (group A to D) Table 1. As shown in Table 1, the time of GSE supplementation (from hatch (one day of age) to week 40) versus from 28 days (week 4) to week 40) significantly affected body weight in the growing and before laying periods whereas no significant effect on the fattening was found for this parameter whatever the periods (starting to laying period). Body weight was significantly reduced in D group as compared to other groups (Fig 1A, Table 1). During the laying period, the body weight of C and D group was significantly lower than control groups (3539.7 ± 26.62, 3499.04 ± 32.32 *vs* 3640.61 ± 26.71, *P* < 0.05). Moreover, we noted a significant effect of the time of GSE supplementation only for the starting period for the feed conversion. Indeed, during this period an increase of the feed conversion (Fig 1B, Table 1) for the D group compared to the control group (2.2 ± 0.07 *vs* 2.5 ± 0.03, *P* = 0.001) was observed. Concerning the fattening level (Fig 2, Table 1), we did not observe any difference for the starting and laying period but a reduction (*P* < 0.05) was observed in the D group during the growth and before the laying. Interestingly, this difference was noticed between C and D group (growing: 3.26 ± 0.04 *vs* 3.07 ± 0.05, *P* = 0.01; before laying 4.41 ± 0.08 *vs* 4.08 ± 0.08, *P* = 0.03).

**Table 1:** Effect of time (start at hatch or start at 4 week-old until 40 week-old) and level (A: No supplementation, B and C: Supplementation at 0.5% and 1% of the total diet composition, respectively, starting at 4 week-old until 40 week-old, respectively, and D: Supplementation at 1% of the total diet composition starting at hatch until 40 week-old) of GSE dietary supplementation on live body weight, fattening level (as determined by ultrasound) and feed conversion in hens. Results are presented as lsmeans ± SEM. P values of the effects of the stage when the supplementation is applied (TimeSuppl) and diet were considered as significant if *P* < 0.05. Different individual letters (a, b and c) in superscript indicate a significant effect of the diet.

Period	Diet	Body weight (g)	Fat (mm)	Feed conversion	Feed intake (g/day)
**0 to 4**	A	294.17 ± 13.39 ^a^	2.76 ± 0.06	2.15 ± 0.07	35.29 ± 0.16
**Starting**	B	303.3 ± 15.03 ^a^	2.81 ± 0.06	2.12 ± 0.1	35.44 ± 0.09
	C	297.94 ± 14.38 ^a^	2.74 ± 0.07	2.12 ± 0.06	35.45 ± 0.1
	D	244.93 ± 12.93 ^b^	2.61 ± 0.08	2.55 ± 0.03	34.64 ± 0.62
*P*	TimeSuppl	0.15	0.14	**0.0005**	0.07
*P*	Diet ABCD	**0.0193**	0.23	-	0.17
*P*	Diet ABC	-	-	0.93	-
*P*	Diet AD	-	-	**0.0011**	-
**5 to 17**	A	1160.68 ± 18.12	3.2 ± 0.04 ^a, b^	4.64 ± 0.06	57.62 ± 2.45
**Growing**	B	1160.24 ± 26.19	3.18 ± 0.04 ^a, b^	4.66 ± 0.05	57.62 ± 2.45
	C	1127.91 ± 19.62	3.26 ± 0.04 ^a^	4.65 ± 0.04	57.62 ± 2.45
	D	1057.87 ± 21.36	3.07 ± 0.05 ^b^	4.87 ± 0.11	57.62 ± 2.45
*P*	TimeSuppl	**<0.0001**	0.13	0.07	-
*P*	Diet ABCD	-	**0.0118**	0.17	-
*P*	Diet ABC	0.28	-	-	-
*P*	Diet AD	**<0.0001**	-	-	-
**18 to 23**	A	2126.73 ± 28.06	4.29 ± 0.08 ^a, b^	5.35 ± 0.49	97.17 ± 5.45
**Before laying**	B	2119.8 ± 32.94	4.26 ± 0.08 ^a, b^	5.02 ± 0.19	97.17 ± 5.45
	C	2108.23 ± 31.63	4.41 ± 0.08 ^a^	4.92 ± 0.09	97.17 ± 5.45
	D	1984.38 ± 34.46	4.08 ± 0.08 ^b^	5.34 ± 0.18	97.17 ± 5.45
*P*	TimeSuppl	**0.018**	0.27	0.48	-
*P*	Diet ABCD	-	**0.0316**	0.69	-
*P*	Diet ABC	0.94	-	-	-
*P*	Diet AD	**0.0083**	-	-	-
**24 to 40**	A	3640.61 ± 26.71 ^a^	5.66 ± 0.06	-	140.76 ± 2.93
**Laying**	B	3583.44 ± 29.26 ^a, b^	5.57 ± 0.06	-	140.76 ± 2.93
	C	3539.7 ± 26.62 ^b^	5.56 ± 0.06	-	140.76 ± 2.93
	D	3499.04 ± 32.32 ^b^	5.52 ± 0.06	-	140.76 ± 2.93
*P*	TimeSuppl	0.1	O.88	-	-
*P*	Diet ABCD	**0.0056**	0.43	-	-
*P*	Diet ABC	-	-	-	-
*P*	Diet AD	**-**	-	-	-

### Effects of GSE supplementation on TAS, TOS and Oxidative Stress Index (OSI)

**Fig 3:**
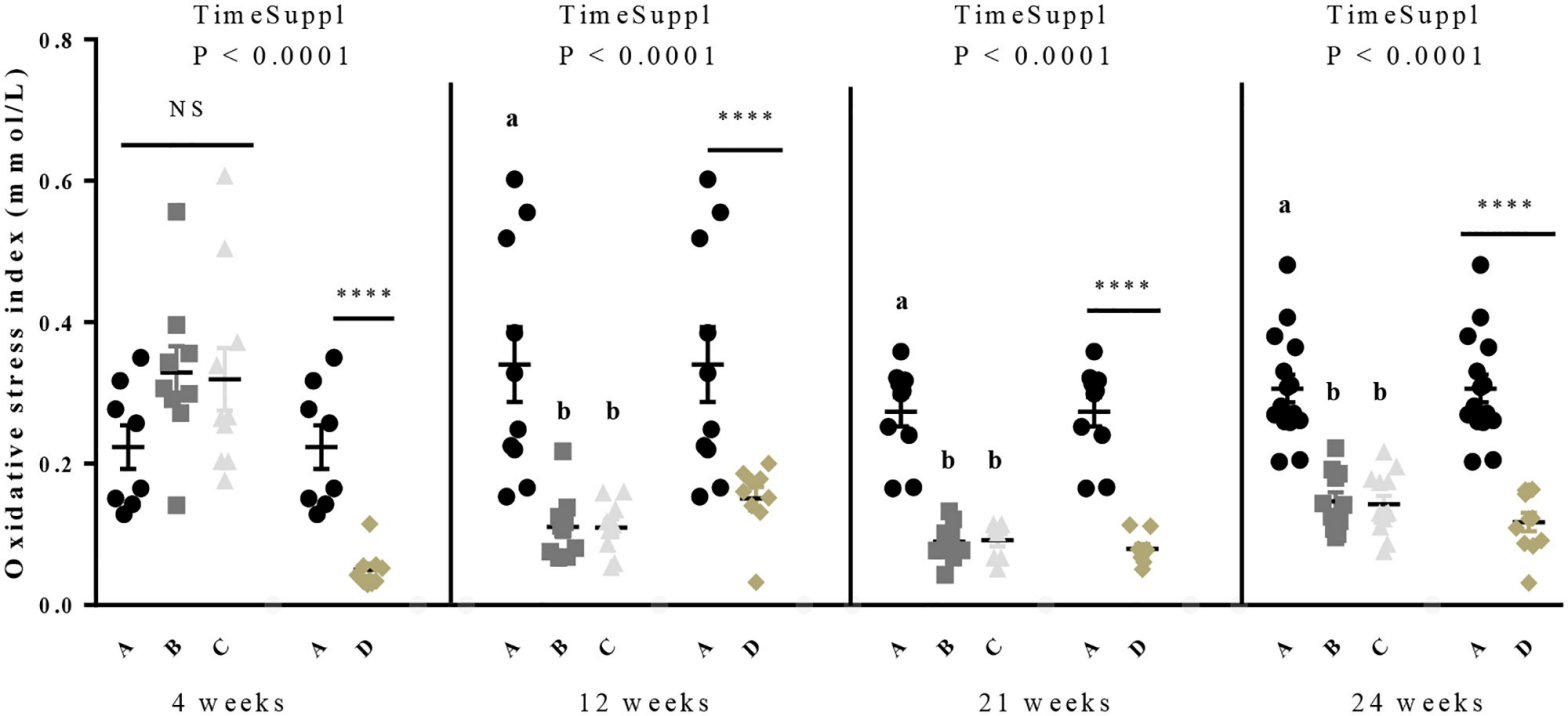
Oxidative Stress Index (OSI) in broiler hens fed with different concentrations of GSE dietary supplementation. Blood samples were collected at 4, 12, 21 and 24 weeks, before diet distribution. The OSI was calculated by the Total Oxidant Status (TOS) divided by the Total Antioxidant Status (TAS) A: animals fed with control diet without GSE supplementation (n = 10), B and C: animals supplemented with GSE at 0.5% and 1% of the total diet composition, respectively, starting at 4 week-old until 40 week-old (n = 10) and D: supplementation at 1% of the total diet composition starting at hatch until 40 week-old (n = 10). Results are presented as lsmeans ± SEM. P values of the effects of the stage when the supplementation is applied (TimeSuppl) and diet were considered as significant if *P* < 0.05. Different individual letters (a, b and c) indicate a significant effect of the diet. **** *P* < 0.0001 (diet effect).

As shown in the Table 2, a significant effect of the time of GSE supplementation was observed for both TOS and OSI whatever the period whereas it was significant only for the growth and laying period for the TAS parameter. Interestingly, during the growth period, we showed a decrease for the D group compared to the control group (0.85 ± 0.06 *vs* 0.63 ± 0.04, *P* = 0.01) (Fig 3). Then, during the laying period, from 21 to 40 weeks, the TAS decreased for all GSE supplemented groups compared to the control group (0.87 ± 0.03 *vs* 0.77 ± 0.02, 0.76 ± 0.02, 0.73 ± 0.03, *P* < 0.005). The TOS significantly decreased for all GSE supplemented groups during the starting, growth and laying periods (*P* < 0.001). The OSI was decreased (Table 2, Fig 3) in D group compared to the control at each period (starting: 0.27 ± 0.05 *vs* 0.05 ± 0.009, *P* = 0.0006; growth: 0.35 ± 0.03 *vs* 0.13 ± 0.01 *P* < 0.0001; before laying: 0.27 ± 0.02 *vs* 0.08 ± 0.007, *P* < 0.0001; laying: 0.31 ± 0.01 *vs* 0.12 ± 0.007, *P* < 0.0001). It was also reduced in B and C groups as compared to control in growing, before laying and laying period (growth: 0.35 ± 0.03 *vs* 0.22 ± 0.03, 0.27 ± 0.05 *P* = 0.03; before laying: 0.27 ± 0.02 *vs* 0.09 ± 0.008, 0.21 ± 0.12, *P* < 0.0001; laying: 0.31 ± 0.01 *vs* 0.15 ± 0.02, 0.13 ± 0.01, *P* < 0.0001).

**Table 2:** Effect of time (start at hatch or start at 4 week-old until 40 week-old) and level (A: No supplementation, B and C: Supplementation at 0.5% and 1% of the total diet composition, respectively, starting at 4 week-old until 40 week-old and D: Supplementation at 1% of the total diet composition starting at hatch until 40 week-old) of GSE dietary supplementation on Total Antioxidant Status (TAS), Total Oxidant Status (TOS) and Oxidative Stress Index (OSI) in hens plasma. Results are presented as lsmeans ± SEM. P values of the effects of the stage when the supplementation is applied (TimeSuppl) and diet were considered as significant if *P* < 0.05. Different individual letters (a, b and c) in superscript indicate a significant effect of the diet.

Period		TAS (mmol/L)	TOS (mmol/L)	Oxidative stress index
**0 to 4**	A	0.69 ± 0.06	0.16 ± 0.01 ^a^	0.27 ± 0.05
**Starting**	B	0.82 ± 0.07	0.25 ± 0.02 ^b^	0.33 ± 0.04
	C	0.95 ± 0.1	0.25 ± 0.01 ^b^	0.32 ± 0.04
	D	0.82 ± 0.07	0.04 ± 0.004	0.05 ± 0.009
*P*	TimeSuppl	0.22	**<0.0001**	**<0.0001**
*P*	Diet ABCD	0.14	-	-
*P*	Diet ABC	-	**0.0004**	0.57
*P*	Diet AD	-	**<0.0001**	**0.0006**
**5 to 17**	A	0.85 ± 0.06	0.25 ± 0.01 ^a^	0.35 ± 0.03 ^a^
**Growth**	B	0.87 ± 0.06	0.16 ± 0.02 ^b^	0.22 ± 0.03 ^b^
	C	0.64 ± 0.04	0.16 ± 0.02 ^b^	0.27 ± 0.05 ^a, b^
	D	0.63 ± 0.04	0.07 ± 0.008	0.13 ± 0.01
*P*	TimeSuppl	**0.04**	**<0.0001**	**<0.0001**
*P*	Diet ABCD	-	-	-
*P*	Diet ABC	0.18	**<0.0001**	**0.03**
*P*	Diet AD	**0.01**	**<0.0001**	**<0.0001**
**18 to 23**	A	0.9 ± 0.09	0.23 ± 0.01	0.27 ± 0.02 ^a^
**Before laying**	B	0.77 ± 0.06	0.06 ± 0.01	0.09 ± 0.008 ^b^
	C	0.84 ± 0.08	0.17 ± 0.09	0.21 ± 0.12 ^b^
	D	0.88 ± 0.05	0.07 ± 0.01	0.08 ± 0.007
*P*	TimeSuppl	0.38	**0.02**	**<0.0001**
*P*	Diet ABCD	0.61	-	-
*P*	Diet ABC	-	0.06	**<0.0001**
*P*	Diet AD	-	**<0.0001**	**<0.0001**
**24 to 40**	A	0.87 ± 0.03 ^a^	0.26 ± 0.01 ^a^	0.31 ± 0.01 ^a^
**Laying**	B	0.77 ± 0.02 ^b^	0.11 ± 0.01 ^b^	0.15 ± 0.02 ^b^
	C	0.76 ± 0.02 ^b^	0.09 ± 0.01 ^b^	0.13 ± 0.01 ^b^
	D	0.73 ± 0.03	0.08 ± 0.004	0.12 ± 0.007
*P*	TimeSuppl	**0.005**	**<0.0001**	**<0.0001**
*P*	Diet ABCD	-	-	-
*P*	Diet ABC	**0.001**	**<0.0001**	**<0.0001**
*P*	Diet AD	0.08	**<0.0001**	**<0.0001**

### Effects of GSE supplementation on plasma metabolic parameters concentrations

**Fig 4:**
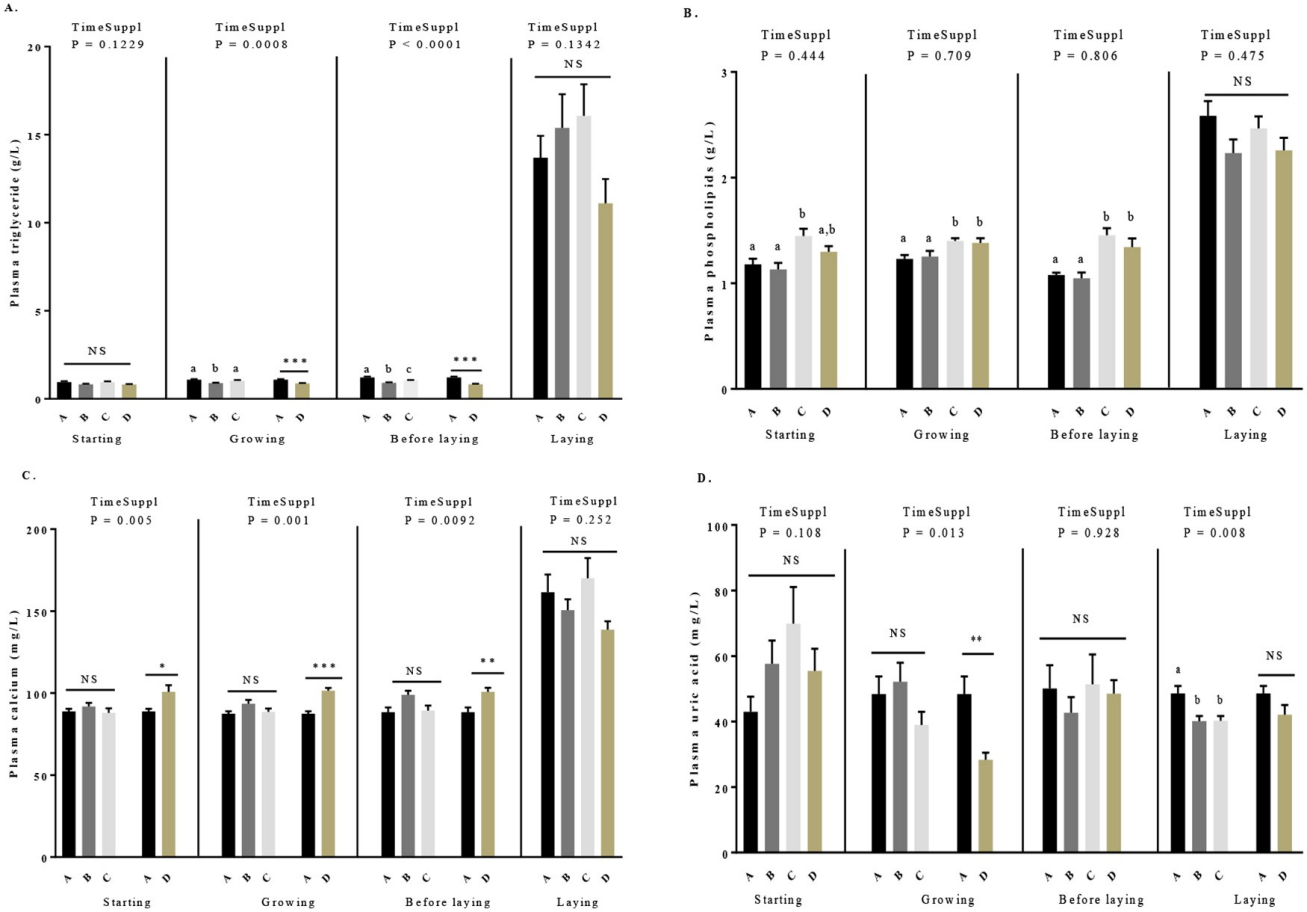
Plasma concentration of triglycerides (A), phospholipids (B), calcium (C) and uric acid (D) in broiler hens fed with different concentrations of GSE dietary supplementation or with a control diet. Blood samples were collected from 4 to 40 weeks, before diet distribution. A: animals fed with control diet without GSE supplementation (n = 10), B and C: animals supplemented with GSE at 0.5% and 1% of the total diet composition, respectively, starting at 4 week-old until 40 week-old (n = 10), and D: supplementation at 1% of the total diet composition starting at hatch until 40 week-old (n = 10). The starting period corresponds to the hatching until the 4th week, the growing period corresponds to the 4th week until 17th week, the « before laying » period corresponds to the 18th week to the 23rd week and the laying period corresponds to the 24th week until the 40th week. Results are presented as lsmeans ± SEM. P values of the effects of the stage when the supplementation is applied (TimeSuppl) and diet were considered as significant if P < 0.05. Different individual letters (a, b and c) indicate a significant effect of the diet. * P < 0.05,** P < 0.005 and *** P < 0.001 (diet effect).

As shown in the Table 3, during the starting period, there was no difference for plasma concentrations of triglycerides (Fig 4A), glucose (S3 Table) and uric acid (Fig 4D). However, we noted significant variations for plasma concentrations of phospholipids (Fig 4B) and calcium (Fig 4C, *P* < 0.05). More precisely, for phospholipids concentration (Fig 4B), no time supplementation effect was observed and we showed an increase in the C group compared to A and B groups (1.45 ± 0.07 *vs* 1.18 ± 0.05 and 1.13 ± 0.06, *P* < 0.005, respectively). For the plasma calcium (Table 3, Fig 4C), a time supplementation effect was noted. The D group had higher plasma calcium concentration as compared to control group (A) (100.77 ± 4.03 *vs* 88.87 ± 1.52, *P* = 0.01).

**Table 3:** Effect of time (start at hatch or start at 4 week-old until 40 week-old) and level (A: No supplementation, B and C: Supplementation at 0.5% and 1% of the total diet composition, respectively, starting at 4 week-old until 40 week-old and D: Supplementation at 1% of the total diet composition starting at hatch until 40 week-old) of GSE dietary supplementation on plasma triglycerides, phospholipids, calcium and uric acid concentrations in hens. Results are presented as lsmeans ± SEM. P values of the effects of the stage when the supplementation is applied (TimeSuppl) and diet were considered as significant if *P* < 0.05. Different individual letters (a, b and c) in superscript indicate a significant effect of the diet.

Periods		Triglycerides (g/L)	Phospholipids (g/L)	Calcium (mg/L)	Uric acid (mg/L)
**0 to 4**	A	0.95 ± 0.05	1.18 ± 0.05 ^a^	88.87 ± 1.52	42.98 ± 4.63
**Starting**	B	0.84 ± 0.03	1.13± 0.06 ^a^	91.82 ± 2.18	52.59 ± 5.66
	C	0.94 ± 0.06	1.45 ± 0.07 ^b^	87.91 ± 2.84	49.99 ± 5.95
	D	0.84 ± 0.03	1.3 ± 0.05 ^ab^	100.77 ± 4.03	49.12 ± 4.71
*P*	TimeSuppl	0.12	0.34	**0.005**	0.11
*P*	Diet ABCD	0.13	**0.003**	**-**	0.57
*P*	Diet ABC	-	-	0.45	-
*P*	Diet AD	-	-	**0.01**	**-**
**5 to 17**	A	1.09 ± 0.04 ^a^	1.24 ± 0.04 ^a^	87.39 ± 1.61	44.32 ± 4.32
**Growth**	B	0.89 ± 0.02 ^b^	1.27 ± 0.06 ^a^	93.59 ± 2.26	45.75 ± 3.85
	C	1.03 ± 0.04 ^a^	1.4 ± 0.03 ^b^	88.55 ± 2.1	38.98 ± 4
	D	0.88 ± 0.02	1.38 ± 0.05 ^b^	101.48 ± 1.72	28.32 ± 2.18
*P*	TimeSuppl	**0.0008**	0.06	**0.001**	**0.01**
*P*	Diet ABCD	-	**0.01**	**-**	-
*P*	Diet ABC	**0.003**	**-**	0.25	0.23
*P*	Diet AD	**0.0002**	**-**	**0.0002**	**0.004**
**18 to 23**	A	1.22 ± 0.05 ^a^	1.08 ± 0.02 ^a^	86.47 ± 3.16	45.37 ± 5.9
**Before laying**	B	0.89 ± 0.02 ^b^	1.05 ± 0.06 ^a^	96.63 ± 3.21	38.96 ± 3.35
	C	1.04 ± 0.03 ^c^	1.45 ± 0.07 ^b^	89.33 ± 3.12	40.46 ± 2.38
	D	0.83 ± 0.03	1.34 ± 0.08 ^b^	100.7 ± 2.5	48.64 ± 2.85
*P*	TimeSuppl	**<0.0001**	0.07	**0.009**	0.92
*P*	Diet ABCD	-	**<0.0001**	**-**	0.33
*P*	Diet ABC	**0.0001**	**-**	0.08	-
*P*	Diet AD	**0.0002**	**-**	**0.002**	**-**
**24 to 40**	A	13.98 ± 1.76	2.55 ± 0.19	163.86 ± 11.32 ^a^	49 ± 2.63 ^a^
**Laying**	B	14.98 ± 2.54	2.19 ± 0.15	150.44 ± 7.6 ^a^	40.28 ± 1.87 ^b^
	C	17.05 ± 2.13	2.5 ± 0.14	181.76 ± 16.5 ^a^	39.94 ± 1.53 ^b^
	D	12.38 ± 1.68	2.31 ± 0.13	140.35 ± 5.57	42.44 ± 3.83
*P*	TimeSuppl	0.13	0.39	0.25	**0.008**
*P*	Diet ABCD	0.32	0.17	**0.01**	**-**
*P*	Diet ABC	-	-	-	**0.008**
*P*	Diet AD	-	-	-	0.09

During the growing period, we did not observe any difference for glucose concentration (S3 Table). For the triglycerides concentrations (Table 3, Fig 4A), we showed a time effect of GSE supplementation. They were reduced in D group as compared to control (0.88 ± 0.02 *vs* 1.09 ± 0.04, *P* = 0.0002) and decreased in B group as compared to control or C group (0.89 ± 0.02 *vs* 1.09 ± 0.04 and 1.03 ± 0.04, *P* = 0.003). For the plasma uric acid (Fig 4D) and calcium concentrations (Fig 4C), a time effect of GSE supplementation was observed. Calcium concentration significantly increased in the D group (101.48 ± 1.72 *vs* 87.39 ± 1.61, *P* < 0.001) while acid uric concentration (Fig 4D) significantly decreased in D group compared the control group (28.32 ± 2.18 *vs* 44.32 ± 4.32, *P* < 0.01). No difference was showed between the A, B and C groups.

During the period “before the laying”, no effect of time GSE supplementation and diet was observed for plasma uric acid (Table 3, Fig 4D) and glucose concentration (S3 Table). For plasma triglycerides concentration (Fig 4A), an effect of time GSE supplementation was noted. A significant reduction was observed in D group as compared to control group (0.83 ± 0.03 *vs* 1.22 ± 0.05, *P* = 0.0002) and between B and C and control group (0.89 ± 0.02, 1.04 ± 0.03 *vs* 1.22 ± 0.05, *P* = 0.0001). For plasma calcium concentration (Fig 4C) an effect of time GSE supplementation whereas no effect of the diet were observed (Table 3). Plasma calcium concentration was significantly increased in D group compared to the control group (100.7 ± 2.5 *vs* 86.47 ± 3.16, *P* = 0.002).

During the laying period, GSE supplementation had no effect on plasma triglycerides (Fig 4A), phospholipids (Fig 4B, Table 3) and glucose concentration (S3 Table). A time GSE supplementation effect was observed for plasma uric acid but not for plasma calcium concentration. Plasma uric acid concentration (Fig 4D) was significantly decreased in B and C group compared to control group (40.28 ± 1.87 and 39.94 ± 1.53 *vs* 49 ± 2.63, *P* < 0.01) but not altered in D group. Plasma calcium concentration (Fig 4C) was significantly lower in D group as compared to other group (A, B and C, 140.35 ± 5.57 *vs* 163.86 ± 11.32, 150.44 ± 7.6 and 181.76 ± 16.5, *P* = 0.01).

### Plasma adipokines concentrations and association with oxidative stress parameters in response to GSE supplementation

**Fig 5:**
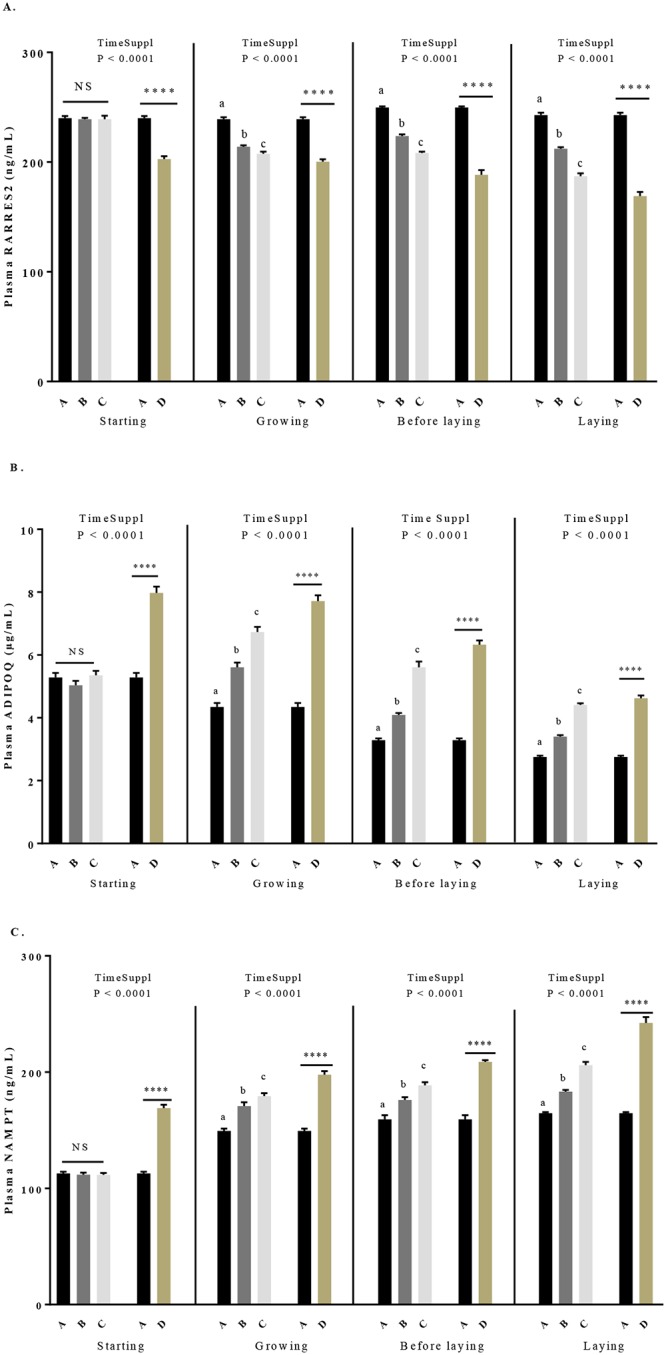
Plasma concentration of RARRES2 (A), ADIPOQ (B) and NAMPT (C) in broiler hens fed with different concentrations of GSE dietary supplementation or with a control diet. A: animals fed with control diet without GSE supplementation (n = 92), B and C: animals supplemented with GSE at 0.5% and 1% of the total diet composition, respectively, starting at 4 week-old until 40 week-old (n = 80), and D: supplementation at 1% of the total diet composition starting at hatch until 40 week-old (n = 72). The starting period corresponds to the hatching until the 4th week, the growing period corresponds to the 4th week until 17th week, the « before laying » period corresponds to the 18th week to the 23rd week and the laying period corresponds to the 24th week until the 40th week. Results are presented as lsmeans ± SEM. P values of the effects of the stage when the supplementation is applied (TimeSuppl) and diet were considered as significant if P < 0.05. Different individual letters (a, b and c) indicate a significant effect of the diet. **** P < 0.0001 (diet effect).

As shown in Table 4, dietary GSE supplementation significantly affected plasma RARRES2 (Fig 5A), ADIPOQ (Fig 5B) and NAMPT (Fig 5C) concentration, from 0 to 40 weeks. More precisely, plasma RARRES2 concentration decreased whereas plasma ADIPOQ and NAMPT concentrations increased according to the concentration of GSE supplementation and the time of supplementation. We showed significant differences between the control and D group (*P* < 0.0001) from the starting period to the laying period and, between the control and B, C groups from the growing to laying period (*P* < 0.0001) for all adipokines.

**Table 4:** Effect of time (start at hatch or start at 4 week-old until 40 week-old) and level (A: No supplementation, B and C: Supplementation at 0.5% and 1% of the total diet composition, respectively, starting at 4 week-old until 40 weeks-old and D: Supplementation at 1% of the total diet composition starting at hatch until 40 weeks-old) of GSE dietary supplementation on plasma adipokine concentrations in hens. Results are presented as lsmeans ± SEM. P values of the effects of the stage when the supplementation is applied (TimeSuppl) and diet were considered as significant if *P* < 0.05. Different individual letters (a, b and c) in superscript indicate a significant effect of the diet.

Periods		RARRES2 (ng/mL)	ADIPOQ (μg/mL)	NAMPT (ng/mL)
**0 to 4**	A	240 ± 2.02	5.28 ± 0.15	112.8 ± 1.45
**Starting**	B	239 ± 1.34	5.04 ± 0.14	111.8 ± 1.61
	C	239 ± 3.3	5.36 ± 0.14	111.7 ± 1.53
	D	202.8 ± 2.58	7.97 ± 0.2	169 ± 2.88
*P*	TimeSuppl	**<0.0001**	**<0.0001**	**<0.0001**
*P*	Diet ABCD	-	-	-
*P*	Diet ABC	0.9417	0.2753	0.8513
*P*	Diet AD	**<0.0001**	**<0.0001**	**<0.0001**
**5 to 17**	A	239 ± 1.82 ^a^	4.35 ± 0.13 ^a^	149.45 ± 1.95 ^a^
**Growing**	B	214 ± 1.2 ^b^	5.61 ± 0.15 ^b^	170.73 ± 3.27 ^b^
	C	207.6 ± 1.87 ^c^	6.73 ± 0.16 ^c^	179.41 ± 2.4 ^c^
	D	200.4 ± 2.17	7.71 ± 0.19	197.8 ± 3.04
*P*	TimeSuppl	**<0.0001**	**<0.0001**	**<0.0001**
*P*	Diet ABCD	-	-	-
*P*	Diet ABC	**<0.0001**	**<0.0001**	**<0.0001**
*P*	Diet AD	**<0.0001**	**<0.0001**	**<0.0001**
**18 to 23**	A	249.8 ± 1.02 ^a^	3.29 ± 0.05 ^a^	159.3 ± 3.51 ^a^
**Before laying**	B	223.7 ± 1.6 ^b^	4.09 ± 0.06 ^b^	176.1 ± 2.28 ^b^
	C	208.2 ± 1.29 ^c^	5.61 ± 0.18 ^c^	188.6 ± 2.72 ^c^
	D	188.4 ± 4.29	6.33 ± 0.13	208.8 ± 1.37
*P*	TimeSuppl	**<0.0001**	**<0.0001**	**<0.0001**
*P*	Diet ABCD	-	-	-
*P*	Diet ABC	**<0.0001**	**<0.0001**	**<0.0001**
*P*	Diet AD	**<0.0001**	**<0.0001**	**<0.0001**
**24 to 40**	A	242.8 ± 2.18 ^a^	2.69 ± 0.07 ^a^	164.63 ± 0.98 ^a^
**Laying**	B	212.1 ± 1.49 ^b^	3.4 ± 0.05 ^b^	183.16 ± 1.55 ^b^
	C	187.1 ± 2.71 ^c^	4.41 ± 0.06 ^c^	206.03 ± 2.79 ^c^
	D	169.1 ± 3.67	4.62 ± 0.09	242.3 ± 5.03
*P*	TimeSuppl	**<0.0001**	**<0.0001**	**<0.0001**
*P*	Diet ABCD	-	-	-
*P*	Diet ABC	**<0.0001**	**<0.0001**	**<0.0001**
*P*	Diet AD	**<0.0001**	**<0.0001**	**<0.0001**

As shown in Table 5, when all group (A to D) and period (starting to laying period) were included in the statistical analyse groups, we observed that the plasma levels of RARRES2 and ADIPOQ were negatively correlated with the live body weight (RARRES2: r = -0.3, *P* < 0.0001; ADIPOQ: r = -0.67, *P* < 0.0001) while plasma NAMPT was positively correlated with this parameter (r = 0.55, *P* < 0.0001). Low significant correlations were observed between plasma TAS and RARRES2 (r = 0.19, *P* = 0.0007) and plasma TAS and ADIPOQ (r = -0.18, *P* = 0.002) and between plasma TOS and ADIPOQ (r = 0.16, *P* = 0.009) and plasma TOS and NAMPT (r = -0.13, *P* = 0.04). In addition, plasma TOS and RARRES2 were positively correlated (r = 0.42, *P* <0.0001) whereas plasma TOS and NAMPT or ADIPOQ were negatively correlated (r = -0.44, *P* < 0.0001; r = -0.13, *P* = 0.01, respectively).

**Table 5:** Pearson correlation coefficients (r) calculated between plasma adipokines and body weight, TAS, TOS and OSI during the period from 4 to 40 weeks, for the groups A (no supplementation), B (supplemented with 0.5% of the total diet composition starting at 4 weeks-old until 40 week-old), C (supplemented with 1% of the total diet composition starting at 4weeks-old until 40 weeks-old) and D (supplemented with 1% of the total diet composition starting at hatch until 40 weeks-old). Values of r and significance of the correlations are indicated on the table. Correlations were considered as significant if *P* < 0.05.

Groups ABCD		RARRES2	ADIPOQ	NAMPT
**Live Body Weight**	r	-0.3	-0.67	0.55
	*P*	**<0.0001**	**<0.0001**	**<0.0001**
**TAS**	r	0.19	-0.18	-0.1
	*P*	**0.0007**	**0.002**	0.09
**TOS**	r	0.02	0.16	-0.13
	*P*	0.75	**0.009**	**0.04**
**OSI**	r	0.42	-0.13	-0.44
	*P*	**<0.0001**	**0.01**	**<0.0001**

### Effects of GSE supplementation on mRNA expression of adipokines and their receptors in adipose tissue, liver and pectoralis major muscle at 40 weeks

As shown in Table 6, in abdominal adipose tissue, mRNA expression of RARRES2 and its three receptors (CMKLR1, GPR1 and CCRL2) was similarly regulated in response to GSE. There was no difference for the B group, however, for C and D groups we observed a significant increase, compared to the control group. However, in the liver, there was no effect of GSE on the mRNA expression of RARRES2 and its receptors. No expression of RARRES2 in pectoralis major muscle and no expression of ADIPOQ in liver and muscle (Table 6) were detected. In the abdominal adipose tissue (Table 6), we observed an increase of the ADIPOQ mRNA expression in the D group compared to the control (14.85 ± 4.43 *vs* 40.74 ± 7.28, *P* < 0.05) whereas the ADIPOQ receptors (ADIPOR1 and ADIPOR2) were not affected by GSE supplementation. In the pectoralis major muscle (Table 6), we assessed the mRNA expression of NAMPT that is considered more a myokine than an adipokines in chicken. The mRNA expression of NAMPT was decreased for all GSE supplemented groups as compared to the control (NAMPT: 2359.16 ± 582.49 *vs* 288.24 ± 77.7, 324.17 ± 134.95 and 50.74 ± 15.5, *P* < 0.001).

**Table 6:** mRNA expression of adipokines (RARRES2, ADIPOQ and NAMPT), their receptors (CMKLR1, CCRL2, GPR1, ADIPOR1, ADIPOR2) and oxidant (NOX 4, NOX 5) or anti-oxidant (SOD, GST) genes in abdominal adipose tissue (AT abd), liver and pectoralis major muscle at 40 weeks. Results are presented as lsmeans ± SEM. P values of the effects of the stage when the supplementation is applied (TimeSuppl) and diet were considered as significant if *P* < 0.05. Different individual letters (a, b and c) in superscript indicate a significant effect of the diet. A: control (n = 10), B and C: supplementation at 0.5% (n = 13) and 1% (n = 10) of the total diet composition, respectively, starting at 4 week-old until 40 week-old and D: supplementation at 1% of the total diet composition starting at hatch until 40 week-old (n = 10).

Tissues	Diet	A	B	C	D	TimeSuppl	Diet ABCD	Diet ABC	Diet AD
**AT abd**	RARRES2	0.49 ± 0.11 ^a^	0.5 ± 0.15 ^a^	1.60 ± 0.18 ^b^	1.89 ± 0.39	**<0.0001**	**-**	**<0.0001**	**0.002**
	CMKLR1	0.74 ± 0.34 ^a^	0.58 ± 0.24 ^a^	2.97 ± 0.41 ^b^	3.27 ± 0.38	**0.002**	**-**	**<0.0001**	**0.0001**
	CCRL2	0.83 ± 0.55 ^a^	1.37 ± 0.83 ^a^	11.89 ± 1.43 ^b^	17.75 ± 3.54	**<0.0001**	**-**	**<0.0001**	**0.0005**
	GPR1	0.17 ± 0.07 ^a^	0.2 ± 0.07 ^a^	0.69 ± 0.09 ^b^	0.87 ± 0.26	**0.0107**	**-**	**<0.0001**	**0.02**
	ADIPOQ	14.85 ± 4.43 ^ab^	7.38 ± 1.83 ^a^	22.29 ± 4.53 ^b^	40.74 ± 7.28	**0.0004**	**-**	**0.03**	**0.006**
	ADIPOR1	0.01 ± 0.003	0.02 ± 0.01	0.03 ± 0.01	0.02 ± 0.01	0.69	0.84	-	-
	ADIPOR2	0.04 ± 0.01	0.02 ± 0.01	0.05 ± 0.02	0.04 ± 0.01	0.99	0.72	-	-
**Liver**	RARRES2	5.41 ± 2.29	11.22 ± 4.33	8.22 ± 1.93	9.65 ± 2.48	0.62	0.78	-	-
	CMKLR1	0.33 ± 0.03	0.53 ± 0.12	0.36 ± 0.04	0.3 ± 0.04	**0.03**	**-**	0.21	0.17
	CCRL2	1.45 ± 0.05	1.49 ± 0.14	1.45 ± 0.09	1.45 ± 0.07	0.6	0.78	-	-
	GPR1	0.44 ± 0.06	0.41 ± 0.08	0.45 ± 0.05	0.3 ± 0.01	0.16	0.07	-	-
	NOX 4	19.88 ± 7.11 ^a^	4.25 ± 0.96 ^b^	3.89 ± 0.76 ^b^	4.74 ± 1.72	**0.002**	**-**	**0.004**	0.07
	NOX 5	3.94 ± 1.4 ^a^	1.14 ± 0.2 ^b^	1.49 ± 0.27 ^b^	2.01 ± 0.39	**0.04**	**-**	**0.01**	0.18
	SOD	19.49 ± 5.05	38.31 ± 6.04	34.07 ± 7.64	134 ± 22.71	**0.0001**	**-**	0.21	**0.002**
	GST	21.81 ± 14.31	18.51 ± 2.73	79.48 ± 31.5	149.3 ± 30.15	**0.04**	-	0.06	**0.003**
**Pectoralis Muscle**	NAMPT	2359.16 ± 582.49 ^a^	288.24 ± 77.7 ^b^	324.17 ± 134.95 ^b^	50.74 ± 15.5	**<0.0001**	**-**	**<0.0001**	**0.0009**
	NOX 4	7662.43 ± 1527.86 ^a^	193.13 ± 56.39 ^b^	98.93 ± 27.52 ^b^	39.57 ± 17.92	**<0.0001**	**-**	**<0.0001**	**0.0009**
	NOX 5	1977.21 ± 577.39 ^a^	92.33 ± 38.47 ^b^	29.17 ± 8.07 ^b^	25.84 ± 9.95	**<0.0001**	**-**	**0.0005**	**0.008**
	SOD	257.82 ± 30.32	140.88 ± 45.11	45.35 ± 17.4	12.32 ± 2.59	**<0.0001**	**-**	0.08	**0.002**
	GST	204.34 ± 39.67	120.11 ± 42.72	170.3 ± 54.09	138.79 ± 42.06	0.52	0.58	-	-

### Effects of GSE supplementation on mRNA expression of oxidant and antioxidant genes in liver and pectoralis major muscle at 40 weeks

In order to explain the molecular mechanism of GSE on the OSI, we next assessed the expression of two pro-oxidants (NOX 4 and NOX 5) and antioxidants (SOD and GST) genes in both liver and pectoralis major muscle. As shown in Table 6, in the liver, the expression of NOX 4 and NOX 5 significantly decreased in B and C groups as compared to the control (A group) (NOX 4: 19.88 ± 7.11 *vs* 4.25 ± 0.96 and 3.89 ± 0.76, *P* < 0.01; NOX 5: 3.94 ± 1.4 *vs* 1.14 ± 0.2 and 1.49 ± 0.27, *P* < 0.05). However, the expression did not change for the D group but for the antioxidant genes, SOD and GST, the expression was higher only in this group, compared to the control group (SOD: 19.49 ± 5.05 *vs* 134 ± 22.71, *P* < 0.01; GST: 21.81 ± 14.31 *vs* 149.3 ± 30.15, *P* < 0.01). In the pectoralis muscle (Table 6), the expression of NOX4 and NOX 5 were reduced for all supplemented groups (NOX 4: 7662.43 ± 1527.86 *vs* 193.13 ± 56.39, 98.93 ± 27.52 and 39.57 ± 17.92, *P* < 0.001; NOX 5: 1977.21 ± 577.39 *vs* 92.33 ± 38.47, 29.17 ± 8.07 and 25.84 ± 9.95, *P* < 0.001). There was no difference for the expression of GST. Interestingly, we observed a reduction of the SOD expression only in the D group as compared to the control (257.82 ± 30.32 *vs* 12.32 ± 2.59, *P* < 0.01).

### Effect of maternal dietary GSE supplementation on live body weight, mortality and OSI of the progeny

**Fig 6:**
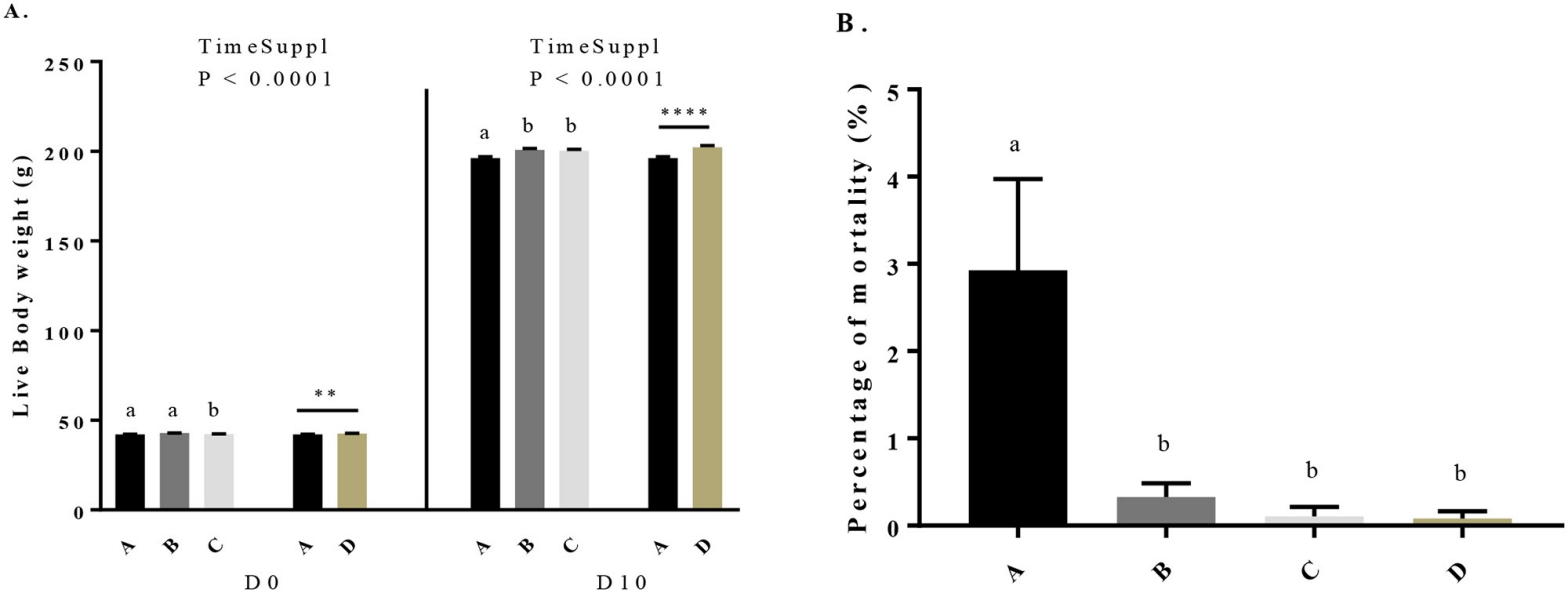
Body weight at the hatching (D0) and after 10 days of age (D10) (A) and mortality level (B) of offsprings from broiler hens fed with different concentrations of GSE dietary supplementation or with a control diet. A: chicks from animals fed with control diet without GSE supplementation (n = 1173), B and C: chicks from animals supplemented with GSE at 0.5% (n = 1159) and 1% (n = 1114) of the total diet composition, respectively, starting at 4 week-old until 40 week-old, and D: chicks from animals fed with GSE supplementation at 1% of the total diet composition starting at hatch until 40 week-old (n = 804). Results are presented as lsmeans ± SEM. P values of the effects of the stage when the supplementation is applied (TimeSuppl) and diet were considered as significant if P < 0.05. Different individual letters (a, b and c) indicate a significant effect of the diet. ** *P* < 0.005 and **** *P* < 0.0001 (diet effect).

**Fig 7:**
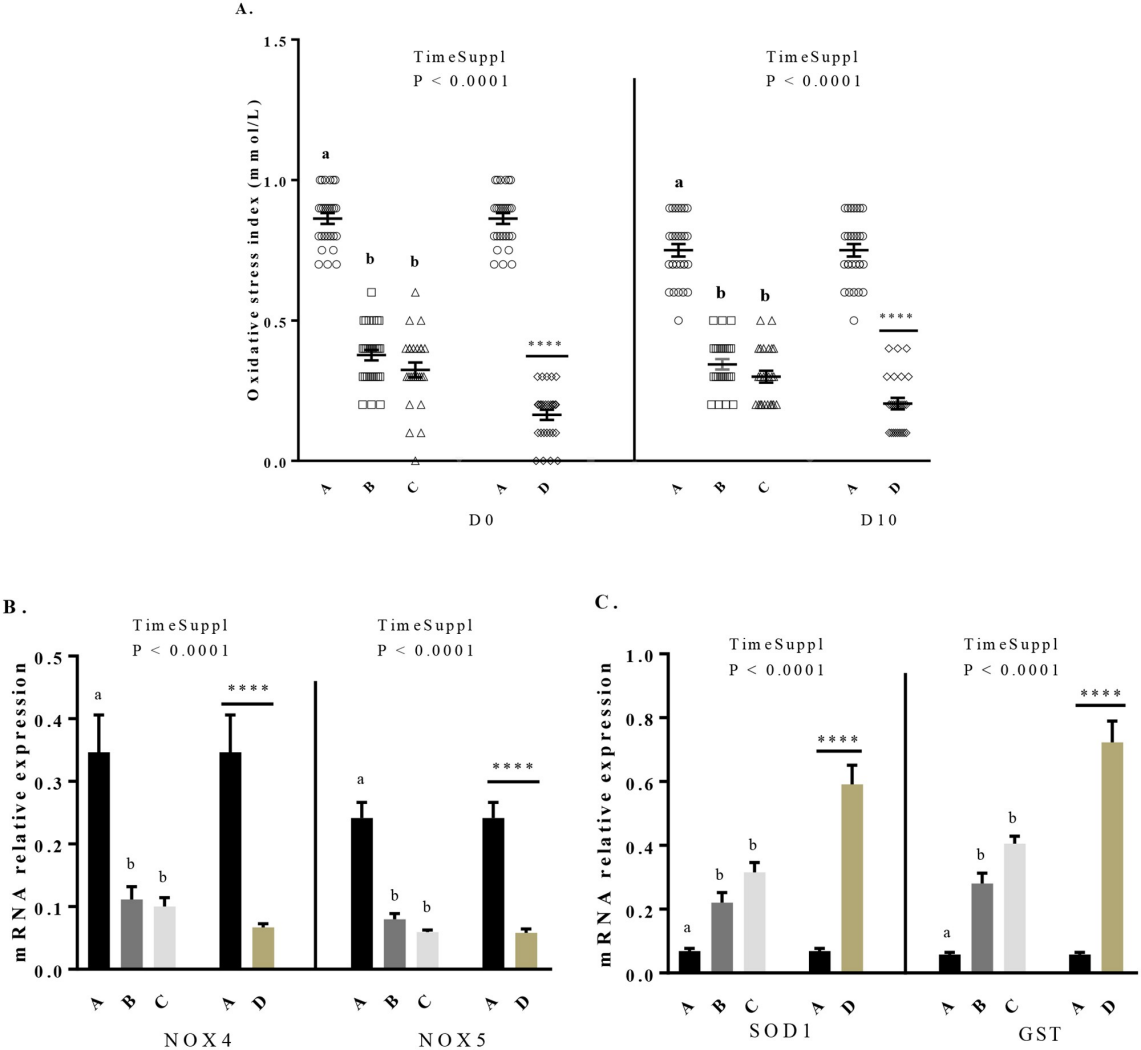
Variations of the offsprings oxidative stress index (A) at the hatching (D0) and after 10 days of age (D10) and mRNA expression of oxidant genes (B) and antioxidant genes (C) in the liver of the chicks after 10 days of age. A: chicks from animals fed with control diet without GSE supplementation (n = 10), B and C: chicks from animals supplemented with GSE at 0.5% and 1% of the total diet composition, respectively, starting at 4 week-old until 40 week-old (n = 10), and D: chicks from animals fed with GSE supplementation at 1% of the total diet composition starting at hatch until 40 week-old (n = 10). Results are presented as lsmeans ± SEM. P values of the effects of the stage when the supplementation is applied (TimeSuppl) and diet were considered as significant if *P* < 0.05. Different individual letters (a, b and c) indicate a significant effect of the diet. **** *P* < 0.0001 (diet effect).

We assessed the live body weight of the chicks at the hatching and after 10 days of age (D10). The percentage of mortality was assessed during this same period. All chicks were fed with a starting diet without GSE supplementation. As shown in Fig 6A, at the hatching, the live body weight increased in chicks from C and D hens groups. Then, at D10, the live body weight of all maternal GSE dietary supplemented groups was higher (196.2 ± 0.81 *vs* 200.8 ± 0.86, 200.2 ± 0.92 and 202.3 ± 0.96, *P* < 0.05) than control group (chicks from control hens (group A)). We also dissected the abdominal and subcutaneous adipose tissue of 40 chicks from hens of different groups at the hatching and day 10 and we observed no effect of the dietary maternal GSE supplementation (S4 Table). However, the maternal dietary GSE supplementation decreased the percentage of mortality in offsprings (*P* < 0.001) (Fig 6B). Concerning the OSI (Fig 7A), the maternal dietary GSE supplementation reduced the OSI at the hatching (0.863 ± 0.02 *vs* 0.378 ± 0.02, 0.324 ± 0.03 and 0.164 ± 0.02, *P* < 0.0001) and at D10 (0.75 ± 0.02 *vs* 0.344 ± 0.02, 0.3 ± 0.02 and 0.204 ± 0.02, *P* < 0.0001). Consistent with these data, we determined the mRNA expression of oxidant (Fig 7B) and antioxidant (Fig 7C) genes in liver at D10. The mRNA expression of oxidant genes, NOX 4 and NOX 5, decreased for all supplemented groups compared to the control group (NOX 4: 0.346 ± 0.06 *vs* 0.111 ± 0.02, 0.1 ± 0.01 and 0.07 ± 0.006, *P* < 0.001; NOX 5: 0.241 ± 0.03 *vs* 0.08 ± 0.009, 0.06 ± 0.003 and 0.06 ± 0.006, *P* < 0.001). Moreover, the mRNA expression of SOD and GST (Fig 7C) increased for all supplemented groups (SOD: 0.069 ± 0.008 *vs* 0.221 ± 0.03, 0.315 ± 0.03 and 0.591 ± 0.06, *P* < 0.001; GST: 0.058 ± 0.007 *vs* 0.28 ± 0.03, 0.406 ± 0.02 and 0.723 ± 0.07, *P* < 0.0001) as compared to the control group. Finally, at hatching, the percentage of male and female was unchanged between chicks from control hens and those from hens dietary supplemented with GSE.

## Discussion

This present study shows that a GSE maternal dietary supplementation reduces plasma and tissue oxidative stress associated to modulation of plasma and tissue adipokines expression without affecting food intake and fattening in reproductive hens. Furthermore, a 1% GSE maternal dietary supplementation increased offspring live body weight and reduced mortality and oxidative stress suggesting a beneficial transgenerational effect.

In our experiment, we showed that a GSE supplementation (1%) performed very early at hatch or lately at the growing period (week 4) did not affect food intake and fattening. However, we observed a significant reduction in the body weight only in the group supplemented at hatch demonstrating a time GSE supplementation effect. In rats, it has been reported that flavanols found in abundance in GSE reduces food intake, and consequently live body weight [37]. This reduction of body weight could be explained by a lipolytic effect [38–40]. Indeed, proanthocyanidins regulate lipid synthesis and degradation through the activation of β-oxidation, which contributes to decreasing lipid accumulation in adipose tissues [17]. Several studies have reported a decrease in the plasma triglycerides in response to dietary GSE supplementation [41–43]. In hens in the present study we did not observe any significant effect on the food intake and fattening. Only a reduction in body weight was observed in the animals supplemented with 1% GSE at one day of age (D group of animal). In humans, a recent meta‐analysis on five studies showed no significant effect on body weight after a treatment with GSE [44]. The species but also the concentration of GSE, the manner of administration (mixed with the diet and free access or gavage (intragastric administration) and the dose used and the time of supplementation could explain this discrepancy. However, in avian species, our data are in good agreement with the literature. Indeed the findings of Abu Hafsa and Ibrahim [45] on the effect of a dietary polyphenol‐rich grape seed (1, 2 and 4% of the diet composition for 42 days) in broiler chicks reported no effect on feed consumption. The negative effect of GSE on live body weight in D group compared to control animals could be explained by a tendency to lower adipose tissue that is associated to significant lower triglyceride plasma concentration. Since this latter result was observed only in animals supplemented at hatch (D group) and not at week 4 (C group) it means that the timing of GSE supplementation can differently affect the growth performance in hens.

Grape seed (Vitis vinifera) extracts are known to contain polyphenols with high antioxidant capacity compared to vitamin E [46, 47]. Thus, it is considered to be a safe and effective antioxidant compound. Some evidence indicates that very often overproduction of free radicals, compromised antioxidant defences and oxidative stress are the leading causes of the detrimental consequences of stress in poultry [48]. The subsequent management practice of feed restriction of reproductive hens leads to chronic hunger and stress [4]. So, there is a challenge in hens to develop a system of optimal antioxidant supplementation to maintain effective antioxidant defences and redox balance in the body. Oxidative stress changes the balance between oxidants and antioxidants either by accumulation of ROS and/or depletion of antioxidants. In general, measuring only one of the oxidant or antioxidant parameters does not provide proper information about the oxidative status. For this reason, in our study we determined both TOS and TAS of plasma. Also, we used OSI as another indicator of oxidative stress. OSI is the ratio of TOS to TAS and it was proposed to reflect the oxidative status more accurately than TOS [36]. Whatever the dose (0.5 or 1% of the diet composition) and the period (at hatch or growing) of GSE supplementation, we observed a significant decrease in the plasma oxidative stress index. This was mainly due to a strong reduction in the plasma TOS that was associated to a decrease in the NOX (NADPH oxidase) 4 and 5 mRNA expression in both pectoralis muscle and liver for each period (starting to laying period). NOX are enzymes that catalyse the conversion of O2 to superoxide (O2−) that can be converted by SOD (superoxide dismutase) to the non- radical species hydrogen peroxide (H2O2). In avian species SOD is a crucial element of the first level of antioxidant defence in the cell [49]. Glutathione S-transferase (GST) is another enzyme that plays an important role in protecting cells and tissues from oxidative damage [50]. Surprisingly, the plasma TAS was not increased whereas SOD and GST mRNA expression were significantly increased in liver but not in pectoralis muscle. Thus, dietary GSE supplementation has beneficial effects in hens by reducing plasma oxidative stress.

In mammals, adipokines are involved in the regulation of several physiological processes [51, 52] including the control of the oxidative stress. Indeed, some reports indicate that NADPH oxidase overexpression and activity could be also related to adipokine imbalance. For example, in ADIPOQ -/- mice, an overexpression of NADPH oxidase subunits has been observed in heart and kidneys strongly showing that ADIPOQ could downregulate superoxide anion production [53]. Moreover, it has been demonstrated that some polyphenols present in GSE such as monomeric procyanidin catechin were inducers of ADIPOQ expression and secretion in the adipocyte cell line3T3-L1 [54]. Thus, GSE treatment through modulation of plasma adipokines could indirectly regulate oxidative stress. Here, we measured plasma levels of three adipokines (ADIPOQ, RARRES2 and NAMPT) that have been the best described in chicken [55] and we determined a correlation between plasma adipokines and oxidative stress parameters. We did not analyse leptin since its expression and its role in birds are still a debate [56]. We observed that plasma RARRES2 and ADIPOQ were negatively correlated with live body weight whereas the opposite was observed for the plasma NAMPT. In a previous study we showed that plasma RARRES2 was significantly associated to fattening in reproductive hens [55]. Furthermore, NAMPT has been described as more a myokine than an adipokine [55, 56]. Here, we showed that dietary GSE supplementation decreases plasma RARRES2 whereas it increases plasma ADIPOQ and NAMPT in each period (from starting to laying period). Thus, GSE can modulate plasma adipokines without significant variation of fattening. We determined mRNA expression of adipokines and their receptors in the main producers tissue in chicken (RARRES2 in liver and adipose tissue, ADIPOQ in adipose tissue and NAMPT in pectoralis muscle). We observed that the GSE effects on plasma adipokines were not always positively associated to variations of mRNA expression of these adipokines suggesting another source of production or a post-transcriptional regulation. However, interestingly we showed that plasma RARRES2 was positively whereas ADIPOQ and NAMPT were negatively associated with the OSI. Thus, plasma adipokines in response to GSE could participate to the oxidative stress regulation in chicken.

The effects of maternal dietary GSE supplementation on the progeny has never been investigated in chicken. In mammals, maternal intake of grape seed procyanidins during pregnancy and lactation in rats fed with a high fat diet increases adiposity and improves the plasma inflammatory profile [22]. Furthermore, it promotes lipid oxidation in the muscle of male offspring in adulthood [21]. Here, we showed that maternal dietary GSE supplementation increases live body weight of offspring and reduces the mortality level without affecting the amount of fat tissue. This latter result could be explained by the reduction of the plasma oxidative stress index. In mammals, oxidative stress during the pregnancy can influence the progeny. It plays a role in fetal programming of cardiovascular disease [57]. Epidemiologic and experimental animal studies reported that placenta appears to play a central role in fetal programming [58]. It is noted that mammals differ from birds in mother-fetus interaction. Maternal factors influence the fetal development via placenta in mammals, while hens deposit nutrients and regulatory signals in the egg. It is well known that the composition of egg yolk largely dependent on the maternal nutrition plays a crucial role in the embryo development. Eggs can be enriched with antioxidants or some unsatured fatty acids by manipulation of poultry feed. For example, a diet for hens supplemented with high-polyphenols level from extra-virgin olive oil can improve the fatty acid quality of egg-yolk [59]. So, modification of the egg composition by dietary maternal GSE supplementation could explain the beneficial effect observed in the progeny in our study. Finally, it will be interesting to determine whether such transgenerational effects of maternal dietary GSE supplementation involve epigenetic gene regulation.

## Conclusions

GSE maternal dietary supplementation reduces plasma and tissue oxidative stress associated to modulation of plasma and tissue adipokines without affecting fattening in reproductive hens. A 1% GSE maternal dietary supplementation increased offspring growth performance and reduced mortality and oxidative stress suggesting a beneficial transgenerational effect. Taken together, our data suggest the possibility of using dietary maternal GSE to improve growth performance of the progeny. However, more experiments are necessary to investigate the effect of sequential maternal dietary GSE supplementation for a short time at specific period such as pre-laying on the growth and laying performance in adult offspring.

## Supporting information

S1 Fig Description of the *in vivo* protocol. From one to 28 days of age (week 4), 324 female breeder chicks received an *ad libitum* diet (free access to food), called a starting control diet or a starting diet supplemented with 1% of the total diet composition with GSE (D; n = 72 animals). At 28 days of age (week 4), animals from control group were segregated into three groups. The first group called A (n = 92 animals) received a control growing diet, the second group called B (n = 80 animals) and the third group called C (n = 80 animals) received the growing diet supplemented with GSE with a concentration of 0.5% and 1% of the total diet composition, respectively. The D group received a growing diet supplemented with 1% of the total diet composition with GSE. From 28 days (week 4) to 280 days of age (week 40), these four groups of animals received three different diets: growing diet until 18 week-old, before laying diet from 18 to 21 weeks and laying diet from 22 to 40 week). The GSE supplementation for B, C and D groups was maintained until 40 week-old.
(TIF)

S1 Table Oligonucleotide primer sequences. (DOCX)

S2 Table Composition of the diet. (A: no supplementation, B and C: supplementation at 0.5% and 1% of the total diet composition, respectively, starting at 4 week-old until 40 week-old, and D: supplementation at 1% of the total diet composition, starting at hatch until 40 week-old).
(DOCX)

S3 Table Effect of time (start at hatch or start at 4 week-old until 40 week-old) and level (A: No supplementation, B and C: Supplementation at 0.5% and 1% of the total diet composition, respectively, starting at 4 week-old until 40 week-old and D: Supplementation at 1% of the total diet composition starting at hatch until 40 week-old) of GSE dietary supplementation on plasma glucose concentrations in hens. Results are presented as lsmeans ± SEM. P values of the effects of the stage when the supplementation is applied (TimeSuppl) and diet were considered as significant if *P* < 0.05. Different individual letters (a, b and c) in superscript indicate a significant effect of the diet.
(DOCX)

S4 Table Weight of subcutaneous and abdominal fat, at the hatching and 10 days of age, in offsprings from broiler hens fed with different dietary GSE supplementations or with a control diet. A: chicks from animals fed with control diet without GSE supplementation (n = 40), B and C: chicks from animals supplemented with GSE at 0.5% (n = 40) and 1% (n = 40) of the total diet composition, respectively, starting at 4 week-old until 40 week-old, and D: chicks from animals fed with GSE supplementation at 1% of the total diet composition starting at hatch until 40 week-old (n = 40). Results are presented as lsmeans ± SEM. *P* values of the effects of the stage when the supplementation is applied (TimeSuppl) and diet were considered as significant if *P* < 0.05. Different individual letters (a, b and c) in superscript indicate a significant effect of the diet. D: day. At hatching, abdominal fat was absent.
(DOCX)
