# Supplementary figures and images for: A grape seed extract maternal dietary supplementation in reproductive hens reduces oxidative stress associated to modulation of plasma and tissue adipokines expression and improves viability of offsprings
Source: PLoS One. 2020 Apr 13;15(4):e0231131. doi: 10.1371/journal.pone.0231131 (PMC7153862; doi:10.1371/journal.pone.0231131)

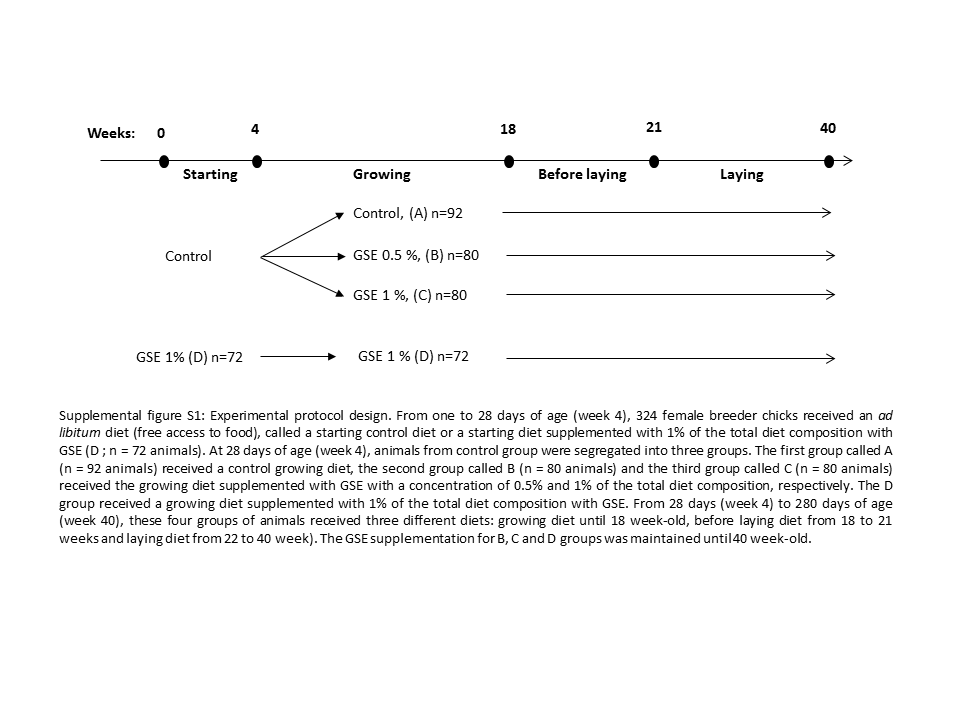

Supplement: S1 Fig — From one to 28 days of age (week 4), 324 female breeder chicks received an ad libitum diet (free access to food), called a starting control diet or a starting diet supplemented with 1% of the total diet composition with GSE (D; n = 72 animals). At 28 days of age (week 4), animals from control group were segregated into three groups. The first group called A (n = 92 animals) received a control growing diet, the second group called B (n = 80 animals) and the third group called C (n = 80 animals) received the growing diet supplemented with GSE with a concentration of 0.5% and 1% of the total diet composition, respectively. The D group received a growing diet supplemented with 1% of the total diet composition with GSE. From 28 days (week 4) to 280 days of age (week 40), these four groups of animals received three different diets: growing diet until 18 week-old, before laying diet from 18 to 21 weeks and laying diet from 22 to 40 week). The GSE supplementation for B, C and D groups was maintained until 40 week-old. (TIF) [file pone.0231131.s001.tif]
