# Supplementary material for: A grape seed extract maternal dietary supplementation in reproductive hens reduces oxidative stress associated to modulation of plasma and tissue adipokines expression and improves viability of offsprings
Source: PLoS One. 2020 Apr 13;15(4):e0231131. doi: 10.1371/journal.pone.0231131 (PMC7153862; doi:10.1371/journal.pone.0231131)
Supplement: S1 Table — (DOCX) [file pone.0231131.s002.docx]

|  | **Starting** | | **Growing** | | | | **Before laying** | | | | **Laying** | | | |
| --- | --- | --- | --- | --- | --- | --- | --- | --- | --- | --- | --- | --- | --- | --- |
| **Weeks** | **0 to 4** | | **5 to 18** | | | | **19 to 21** | | | | **22 to 40** | | | |
| **Diet** | **A** | **D** | **A** | **B** | **C** | **D** | **A** | **B** | **C** | **D** | **A** | **B** | **C** | **D** |
| Corn | 39.64 | 39.64 | 36.58 | 36.58 | 36.58 | 36.58 | 49.33 | 49.33 | 49.33 | 49.33 | 54.19 | 54.19 | 54.19 | 54.19 |
| Wheat | 23.86 | 23.86 | 30 | 30 | 30 | 30 | 22.22 | 22.22 | 22.22 | 22.22 | 8 | 8 | 8 | 8 |
| Soybean meal | 29.3 | 29.3 | 3.32 | 3.32 | 3.32 | 3.32 | 14.37 | 14.37 | 14.37 | 14.37 | 13.95 | 13.95 | 13.95 | 13.95 |
| Wheat bran | 0 | 0 | 11.29 | 11.29 | 11.29 | 11.29 | 0 | 0 | 0 | 0 | 0 | 0 | 0 | 0 |
| Soy oil | 2.2 | 2.2 | 1 | 1 | 1 | 1 | 1.02 | 1.02 | 1.02 | 1.02 | 2 | 2 | **2** | **2** |
| Sunflower oil | 1 | 1 | 14 | 14 | 14 | 14 | 8 | 8 | 8 | 8 | 5.8 | 5.8 | 5.8 | 5.8 |
| Sodium Bicarbonate | 0.18 | 0.18 | 0.1 | 0.1 | 0.1 | 0.1 | 0.1 | 0.1 | 0.1 | 0.1 | 0.1 | 0.1 | 0.1 | 0.1 |
| Calcium carbonate | 0.86 | 0.86 | 1.08 | 1.08 | 1.08 | 1.08 | 2.1 | 2.1 | 2.1 | 2.1 | 6.6 | 6.6 | 6.6 | 6.6 |
| Phosphate | 2.06 | 2.06 | 1.77 | 1.77 | 1.77 | 1.77 | 2.06 | 2.06 | 2.06 | 2.06 | 2.2 | 2.2 | 2.2 | 2.2 |
| Salt | 0.3 | 0.3 | 0.29 | 0.29 | 0.29 | 0.29 | 0.29 | 0.29 | 0.29 | 0.29 | 0.28 | 0.28 | 0.28 | 0.28 |
| Methionine DL | 0.1 | 0.1 | 0 | 0 | 0 | 0 | 0.01 | 0.01 | 0.01 | 0.01 | 0.05 | 0.05 | 0.05 | 0.05 |
| Lysine | 0 | 0 | 0.07 | 0.07 | 0.07 | 0.07 | 0 | 0 | 0 | 0 | 0 | 0 | 0 | 0 |
| Mineral premix | 0.5 | 0.5 | 0.5 | 0.5 | 0.5 | 0.5 | 0.5 | 0.5 | 0.5 | 0.5 | 0.5 | 0.5 | 0.5 | 0.5 |
| Soybean | 0 | 0 | 0 | 0 | 0 | 0 | 0 | 0 | 0 | 0 | 6.33 | 6.33 | 6.33 | 6.33 |
| **Grape Seed Extract** | 0 | 1 | 0 | 0.5 | 1 | 1 | 0 | 0.5 | 1 | 1 | 0 | 0.5 | 1 | 1 |

**Supplemental table**: Composition (%) of the diet (A: no supplementation, B and C: supplementation at 0.5% and 1% of the total diet composition, respectively, starting at 4 week-old until 40 week-old, and D: supplementation at 1% of the total diet composition, starting at birth until 40 week-old).
