# Supplementary material for: A grape seed extract maternal dietary supplementation in reproductive hens reduces oxidative stress associated to modulation of plasma and tissue adipokines expression and improves viability of offsprings
Source: PLoS One. 2020 Apr 13;15(4):e0231131. doi: 10.1371/journal.pone.0231131 (PMC7153862; doi:10.1371/journal.pone.0231131)
Supplement: S2 Table — (A: no supplementation, B and C: supplementation at 0.5% and 1% of the total diet composition, respectively, starting at 4 week-old until 40 week-old, and D: supplementation at 1% of the total diet composition, starting at hatch until 40 week-old). (DOCX) [file pone.0231131.s003.docx]

**Supplemental Table S2:** Oligonucleotide primer sequences

| **Gene** | **Product size (bp)** | **Sequence Forward** | **Sequence Reverse** |
| --- | --- | --- | --- |
| *Actin* | 188 | 5'-ACGGAACCACAGTTTATCATC-3' | 5'-GTCCCAGTCTTCAACTATACC-3' |
|  |  |  |  |
| *RPL15* | 194 | 5'-TGTGATGCGTTTCCTCCTTGG-3' | 5'-CCATAGGTTGCACCTTTTGGG-3' |
|  |  |  |  |
| *RARRES2* | 314 | 5'-CGCGTGGTGAAGGATGTG-3' | 5'-CGACTGCTCCCTAAAGAGGAACT-3' |
|  |  |  |  |
| *CMKLR1* | 403 | 5-'CGGTCAACGCCATTTGGT-3' | 5-'GGGTAGGAAGATGTTGAAGGAA-3' |
|  |  |  |  |
| *CCRL2* | 391 | 5'-CACGCAGTGTTTGCTTTAAAAGC-3' | 5'-CAACAGCCCACGTGACAATG-3' |
|  |  |  |  |
| *GPR1* | 165 | 5'-TGTAAAACGCTTTCCCCTTCTCT-3' | 5'-ATGTCAGCAACTTCACGCAGA-3' |
|  |  |  |  |
| *ADIPOQ* | 64 | 5'-ACAGGTGCAGAAGGACCGAG-3' | 5-'AAGACAGAGCCGCTTGCTTG-3' |
|  |  |  |  |
| *ADIPOR1* | 350 | 5'-GAATACACACCGAGACGGGC-3' | 5'-GCCCAAGACGCAGACAATGG-3' |
|  |  |  |  |
| *ADIPOR2* | 345 | 5-'GAGACTGGCAACATCTGGAC-3' | 5'-TGCGATGCCCAGGACACAAA-3' |
|  |  |  |  |
| *NAMPT* | 96 | 5'-GCTTCAGCCCATTTGGTGA-3' | 5'-ATCCCGGAACTGGATCTTTTG-3' |
|  |  |  |  |
| *NOX4* | 96 | 5'-CCTCTGTGCTTGTACTGTGTAG-3' | 5'-GACATTGGAGGGATGGCTTAT-3' |
|  |  |  |  |
| *NOX5* | 78 | 5'-CCATGGCCAGGACTTTCTT-3' | 5'-GAGCGTCTGACTTCTTCTCTTC-3' |
|  |  |  |  |
| *SOD* | 100 | 5'-GGCTTGTCTGATGGAGATCAT-3' | 5'-GCTTGCCTTCAGGATTAAAGTG-3' |
|  |  |  |  |
| *GST* | 98 | 5'-CCATGGCCAGGACTTTCTT-3' | 5'-GAGCGTCTGACTTCTTCTCTTC-3' |
