# Supplementary material for: A grape seed extract maternal dietary supplementation in reproductive hens reduces oxidative stress associated to modulation of plasma and tissue adipokines expression and improves viability of offsprings
Source: PLoS One. 2020 Apr 13;15(4):e0231131. doi: 10.1371/journal.pone.0231131 (PMC7153862; doi:10.1371/journal.pone.0231131)
Supplement: S3 Table — Results are presented as lsmeans ± SEM. P values of the effects of the stage when the supplementation is applied (TimeSuppl) and diet were considered as significant if P < 0.05. Different individual letters (a, b and c) in superscript indicate a significant effect of the diet. (DOCX) [file pone.0231131.s004.docx]

**Supplemental Table S3. Effect of time (start at hatch or start at 4 week-old until 40 week-old) and level (A: no supplementation, B and C: supplementation at 0.5% and 1% of the total diet composition, respectively, starting at 4 week-old until 40 week-old and D: supplementation at 1% of the total diet composition starting at hatch until 40 week-old) of GSE dietary supplementation on plasma glucose concentration in hens.** Results are presented as lsmeans ± SEM. P values of the effects of the stage when the supplementation is applied (TimeSuppl) and diet were considered as significant if P < 0.05. Different individual letters (a, b and c) in superscript indicate a significant effect of the diet.

| **Periods** |  | **Glucose (mg/L)** |
| --- | --- | --- |
| **0 to 4** | A | 2114.02 ± 60.55 |
| **Starting** | B | 2173.86 ± 27.64 |
|  | C | 2124.37 ± 45.46 |
|  | D | 2053.71 ± 54.93 |
| *P* | TimeSuppl | 0.7669 |
| *P* | Diet ABCD | 0.4165 |
| *P* | Diet ABC | - |
| *P* | Diet AD | - |
| **6 to 18** | A | 2219.59 ± 33.95 |
| **Growth** | B | 2151.14 ± 35.96 |
|  | C | 2143.46 ± 32.47 |
|  | D | 2173.33 ± 25.14 |
| *P* | TimeSuppl | 0.3181 |
| *P* | Diet ABCD | 0.3685 |
| *P* | Diet ABC | - |
| *P* | Diet AD | - |
| **18 to 21** | A | 2174.43 ± 40.51 |
| **Before laying** | B | 2101.73± 23.93 |
|  | C | 2094.8 ± 38.93 |
|  | D | 2033.92 ± 36.33 |
| *P* | TimeSuppl | 0.0992 |
| *P* | Diet ABCD | 0.0703 |
| *P* | Diet ABC | - |
| *P* | Diet AD | - |
| **21 to 40** | A | 1942.48 ± 18.96 |
| **Laying** | B | 1936.8 ± 17.66 |
|  | C | 1899.92 ± 19.19 |
|  | D | 1954.08 ± 22.24 |
| *P* | TimeSuppl | 0.2531 |
| *P* | Diet ABCD | 0.2483 |
| *P* | Diet ABC | - |
| *P* | Diet AD | - |
