# Supplementary material for: A grape seed extract maternal dietary supplementation in reproductive hens reduces oxidative stress associated to modulation of plasma and tissue adipokines expression and improves viability of offsprings
Source: PLoS One. 2020 Apr 13;15(4):e0231131. doi: 10.1371/journal.pone.0231131 (PMC7153862; doi:10.1371/journal.pone.0231131)
Supplement: S4 Table — A: chicks from animals fed with control diet without GSE supplementation (n = 40), B and C: chicks from animals supplemented with GSE at 0.5% (n = 40) and 1% (n = 40) of the total diet composition, respectively, starting at 4 week-old until 40 week-old, and D: chicks from animals fed with GSE supplementation at 1% of the total diet composition starting at hatch until 40 week-old (n = 40). Results are presented as lsmeans ± SEM. P values of the effects of the stage when the supplementation is applied (TimeSuppl) and diet were considered as significant if P < 0.05. Different individual letters (a, b and c) in superscript indicate a significant effect of the diet. D: day. At hatching, abdominal fat was absent. (DOCX) [file pone.0231131.s005.docx]

| **Period** | **Diet** | **Subcutaneous fat (g)** | **Abdominal fat (g)** |
| --- | --- | --- | --- |
| **Hatch** | A | 0.46 ± 0.02 | - |
| **D0** | B | 0.45 ± 0.02 | - |
|  | C | 0.44 ± 0.02 | - |
|  | D | 0.44 ± 0.02 | - |
| *P* | TimeSuppl | 0.79 | - |
| *P* | Diet ABCD | 0.88 | - |
| *P* | Diet ABC | - | - |
| *P* | Diet AD | - | - |
| **D10** | A | 1.65 ± 0.1 | 1.13 ± 0.07 |
|  | B | 1.67 ± 0.1 | 1.14 ± 0.09 |
|  | C | 1.6 ± 0.1 | 1.09 ± 0.07 |
|  | D | 1.78 ± 0.1 | 1.03 ± 0.08 |
| *P* | TimeSuppl | 0.46 | 0.6 |
| *P* | Diet ABCD | 0.62 | 0.73 |
| *P* | Diet ABC | - | - |
| *P* | Diet AD | - | - |
|  |  |  |  |

**Table S4. Weight of subcutaneous and abdominal fat, at the hatching and 10 days of age, in offsprings from broiler hens fed with different percentage of dietary GSE supplementation or with a control diet**. A: chicks from animals fed with control diet without GSE supplementation (n = 40), B and C: chicks from animals supplemented with GSE at 0.5% (n = 40) and 1% (n = 40) of the total diet composition, respectively, starting at 4 week-old until 40 week-old, and D: chicks from animals fed with GSE supplementation at 1% of the total diet composition starting at hatch until 40 week-old (n = 40). Results are presented as lsmeans ± SEM. P values of the effects of the stage when the supplementation is applied (TimeSuppl) and diet were considered as significant if P < 0.05. Different individual letters (a, b and c) in superscript indicate a significant effect of the diet. D: day. At hatching, abdominal fat was absent.
